# Instrumentation Strategies for Monitoring Flow in Centrifugal Compressor Diffusers: Techniques and Case Studies

**DOI:** 10.3390/s25247526

**Published:** 2025-12-11

**Authors:** Emilia-Georgiana Prisăcariu, Oana Dumitrescu

**Affiliations:** Romanian Research and Development Institute for Gas Turbines COMOTI, 061126 Bucharest, Romania; emilia.prisacariu@comoti.ro

**Keywords:** centrifugal compressor, diffuser instrumentation, pressure measurements, temperature measurements, unsteady flow diagnostics, particle image velocimetry, pressure-sensitive paint, rotating stall, surge detection, vibration and acoustic monitoring

## Abstract

Monitoring the complex, three-dimensional flow within centrifugal compressor diffusers remains a major challenge due to geometric confinement, high rotational speeds, and strong unsteadiness near surge and stall. This review provides a comprehensive assessment of contemporary instrumentation strategies for diffuser flow characterization, spanning pressure, temperature, velocity, vibration, and acoustic measurements. The article outlines the standards governing compressor instrumentation, compares conventional probes with emerging high-resolution and high-bandwidth sensor technologies, and evaluates the effectiveness of pressure- and temperature-based diagnostics, optical methods, and advanced dynamic sensing in capturing diffuser behavior. Case studies from industrial compressors, research rigs, and high-speed experimental facilities illustrate how sensor layout, bandwidth, and synchronization influence the interpretation of flow stability, performance degradation, and surge onset. Collectively, these examples demonstrate that high-frequency pressure and temperature probes remain indispensable for instability detection, while optical techniques such as PIV, LDV, and PSP/TSP offer unprecedented spatial resolution for understanding flow structures. The findings highlight the growing integration of hybrid sensing architectures, digital acquisition systems, and data-driven analysis in diffuser research. Overall, the review identifies current limitations in measurement fidelity and accessibility while outlining promising paths toward more robust, real-time monitoring solutions for reliable centrifugal compressor operation.

## 1. Introduction

The diffuser plays a crucial role in both axial and centrifugal compressors, as it is responsible for converting the high kinetic energy of the flow leaving the rotor or impeller into static pressure. In axial compressors, the diffuser, often formed by the stator passages, slows down and redirects the flow to the axial direction, ensuring efficient energy transfer and a uniform velocity profile for the subsequent stage [1]. In centrifugal compressors, the diffuser is positioned downstream of the impeller and achieves pressure recovery by decelerating the high-speed radial flow. It can be designed as vaned, vaneless, or a hybrid type, each offering trade-offs between pressure recovery and stability under off-design conditions [2].

Efficient diffuser performance is important for achieving high overall compressor efficiency and maintaining stable operation. Operating under an adverse pressure gradient, the diffuser is susceptible to boundary-layer separation and flow non-uniformities. When flow separation occurs, it leads to increased aerodynamic losses, reduced static pressure rise, and possible flow instabilities such as surge or stall [3]. Furthermore, unmonitored flow distortions can propagate upstream, adversely affecting rotor performance and reducing the compressor’s operating margin. Local temperature or pressure fluctuations within the diffuser can also cause mechanical fatigue or vibration issues. Therefore, accurate and continuous monitoring helps detect performance degradation early, prevents instability, and supports condition-based maintenance strategies.

Instrumentation in centrifugal compressor diffusers provides essential data for understanding and validating aerodynamic performance, particularly in the context of CFD-based design. Measurements typically include total and static pressure, flow angles, temperature, and, in some advanced setups, time-resolved flow dynamics. These measurements form the foundation for evaluating key performance indicators such as pressure recovery, total pressure loss, flow uniformity, and diffuser efficiency.

Validating flow simulations in centrifugal compressor diffusers remains a significant challenge due to persistent instrumentation limitations. Access to critical-flow regions within the diffuser is often restricted, particularly in high-speed rotating machinery, where compact geometries and complex designs leave little room for sensors. This makes it difficult to deploy tools such as pressure probes, hot-wire anemometry, or laser-based diagnostics without interfering with the flow field, potentially introducing measurement uncertainties. Despite the diffuser’s critical role in overall compressor performance, monitoring capabilities remain limited.

In industrial settings, diffuser diagnostics typically rely on a small number of static pressure or temperature sensors, which offer only a partial view of the highly three-dimensional, unsteady flow structures [4]. These sensors often lack the spatial and temporal resolution needed to capture phenomena such as transient flow separation or secondary flows. Harsh operating environments—marked by high temperatures, pressures, and vibrations—further constrain the use of advanced measurement techniques [5]. While laboratory methods like Particle Image Velocimetry (PIV) and Schlieren imaging provide detailed insights, they are not feasible for real-time, in situ monitoring. Additionally, the integration of experimental data with CFD simulations and data-driven models is still limited, restricting the development of accurate, predictive monitoring systems. Addressing these challenges calls for the advancement of sensing technologies, improved data fusion strategies, and broader adoption of digital twin and AI-based approaches to better capture complex flow behavior and anticipate performance degradation.

This paper presents a comprehensive review of instrumentation techniques used in compressor diffusers, highlighting the advantages and limitations of various approaches, and demonstrating their practical relevance through examples from industrial applications and laboratory research.

## 2. General Instrumentation Standards

Reliable compressor performance evaluation depends not only on precise instrumentation but also on adherence to well-established industrial standards. Over the past decades, organizations such as The American Society for Mechanical Engineers—ASME [6], International Stadardisation Organisation—ISO [7], and The American Petroleum Institute—API [8] have developed comprehensive codes and guidelines that define how compressors should be instrumented, tested, and assessed. These standards ensure consistency across test facilities, provide a common basis for contractual guarantees, and establish best practices for flow, pressure, temperature, vibration, and uncertainty measurement. The following section reviews the most relevant standards, outlining their scope, areas of application, and role in both laboratory and industrial environments.

In research and development environments, compressor instrumentation often extends beyond the boundaries defined by formal standards, requiring tailored approaches that balance accuracy, flexibility, and feasibility. Unlike contractual acceptance tests, where compliance with ASME, ISO, or API codes is mandatory, experimental campaigns may prioritize high spatial or temporal resolution, integration of novel sensors, or the ability to capture unsteady and transient phenomena such as surge inception or rotating stall. These objectives frequently demand compromises, such as accepting higher measurement uncertainty in exchange for faster response times, modifying probe geometries to fit within restricted flow passages, or employing custom-built flow meters not covered by existing codes. In such cases, laboratories develop internal procedures, experimental guidelines, and best practices that surpass the prescriptive scope of standards, ensuring consistency and repeatability while enabling innovation. Over time, these practices become an integral part of institutional knowledge, bridging the gap between formal test codes and the practical needs of cutting-edge compressor research.

In Europe, the reference document for compressor performance testing is ISO 5389—Turbocompressors—Performance test code [9], which establishes procedures for measuring and reporting key parameters, such as pressure ratio, efficiency, corrected speed, and mass flow, under defined reference conditions. The standard provides detailed requirements for test arrangements, instrumentation accuracy, and data reduction methods, making it the contractual baseline for acceptance tests in many European industrial sectors, particularly in energy and process applications. While its scope closely mirrors that of ASME PTC 10 [10] used in North America, ISO 5389 is favored in Europe for its global applicability and harmonization with other ISO machinery standards. In practice, both codes serve the same purpose, but ISO 5389 tends to emphasize international compatibility, whereas ASME PTC 10 is rooted in U.S. practice and terminology, with only minor procedural differences separating the two.

In North America, ASME PTC 19.1—Test Uncertainty [11] provides a dedicated engineering framework for quantifying and propagating measurement uncertainties in performance testing, with examples tailored to rotating machinery and compressor applications. In Europe, there is no direct compressor-specific equivalent; instead, laboratories apply the internationally recognized Guide to the Expression of Uncertainty in Measurement (GUM, ISO/IEC Guide 98-3) [12] together with ISO/IEC 17025 [13], CEN/CEN-Guidance on measurement uncertainty [14], and ISO 21748—Guidance for the use of repeatability, reproducibility, and trueness estimates in measurement uncertainty estimation [15] which requires accredited facilities to evaluate and report uncertainties for all test results.

While PTC 19.1 offers detailed, compressor-focused procedures that integrate directly with ASME performance test codes, the European approach is broader, applying general metrological principles across disciplines and requiring adaptation for turbomachinery testing. Methodologically, both systems are aligned in distinguishing two categories of uncertainty. Type A evaluation relies on statistical analysis of repeated measurements: for example, scatter in pressure readings at constant conditions, the standard deviation of mass flow over a dwell period, or fluctuations in torque and temperature during steady operation. Type B evaluation, on the other hand, is based on information other than direct repetition, such as manufacturer specifications, calibration certificates, or expert judgment. In compressor testing, this includes transducer accuracy (e.g., ±0.05% FS), thermocouple class limits (±1.5 K for Type K Class 1), torque meter calibration data, or speed pickup resolution (±1 rpm). These components are combined and propagated through sensitivity coefficients to yield the overall standard uncertainty of the derived results such as pressure ratio, corrected mass flow, and efficiency. This distinction highlights the different philosophies: ASME emphasizes industry-specific codes with worked engineering examples, whereas the European framework builds on general metrology standards, ensuring consistency across all measurement disciplines but leaving the adaptation to the specific test environment in the hands of laboratories.

Worth mentioning here is the ISO 1217—Displacement Compressors [16] which is focused on positive-displacement machines (such as screw or piston compressors). This standard provides requirements for flow, power, and efficiency testing. It is commonly used for industrial air compressors rather than process turbocompressors.

Another important reference is ISO 10439 (Parts 1–4)—Compressors for Petroleum, Petrochemical, and Natural Gas Industries [17], which cites ISO 5389 for performance testing but extends it with additional requirements on packaging, instrumentation, and reliability tailored to oil and gas applications.

In process and petrochemical industries, the minimum mechanical design, instrumentation, and testing requirements are provided by API 617-Axial and centrifugal compressors [18]. These standards mandate the use of ASME PTC 10 or ISO 5389 for performance testing, depending on the origin of the contract, specifying auxiliary instrumentation such as vibration probes, placement of the bearing temperature sensors, and surge control instrumentation. API 672—Packaged Integrally Geared Centrifugal Air Compressors [19] cover smaller packaged machines used for plant or utility air, emphasizing complete package testing, including drive, control, and auxiliary instrumentation, making it relevant to skid-mounted units.

For flow measurement standards, ISO 9300—Critical-Flow Venturi Nozzles [20] specifies geometry and equations for sonic nozzles, the preferred reference for high-accuracy air flow measurement in compressor test rigs. ISO 5167 (Parts 1–4)—Orifice Plates, Nozzles, and Venturi Tubes [21,22,23,24] defines the differential-pressure primary elements. These are mainly used in industrial and field testing where test gases are not suitable for sonic nozzles. ISO 2186—Pressure Signal Transmission lines [25] governs the design and installation of tubing and manifolds between pressure taps and transducers, being crucial for minimizing dynamic distortion in unsteady or surge prone compressor tests.

For temperature measurements and probe standards, the ASME PTC 19.3 TW-Thermowells [26] provides criteria for thermowell design to ensure safe mechanical behavior and minimal measurement bias. It is applied in compressor discharge temperature measurements, where probe strength and response time must be balanced. IEC/ANSI TC [27] standards for thermocouples and RTDs are also referenced for accuracy classes.

The most relevant vibration and protection standards are: API 670—Machinery Protection Systems [28] which established the requirements for vibration, axial position, overspeed, and surge detection instrumentation and is applied universally in critical process compressors, ensuring the protection sensors’ standardization and compatibility with industry-accepted monitoring systems and ISO 2016-1-Vibration of Machines [29] which provides general procedures for measurement and evaluation vibration severity, usually used for setting acceptance levels for both laboratory and field tests.

Although the previous subsection lists the major standards governing compressor instrumentation, their practical differences warrant a short analytical comparison. ASME PTC 10 and ISO 5389 are broadly equivalent in scope, but PTC 10 remains more prescriptive and provides worked engineering examples tailored to rotating machinery, which simplifies uncertainty evaluation and acceptance testing in North American industrial environments. ISO 5389, by contrast, emphasizes harmonization across international machinery standards and offers greater flexibility in test arrangement, but requires laboratories to define many implementation details themselves (such as instrumentation layouts or correction procedures) based on internal practice. A similar distinction applies to ASME PTC 19.1 [11] versus the European metrology framework (GUM, ISO/IEC 17025): PTC 19.1 integrates uncertainty propagation directly into compressor test codes—its strength being clarity and compressor-specific methodology, whereas the GUM-based approach is more general and rigorous but places greater responsibility on the laboratory to adapt generic principles to turbomachinery contexts. API 617 and ISO 10439 extend performance-test requirements with reliability, protection, and auxiliary instrumentation criteria; their strength is comprehensive coverage for oil-and-gas machinery, though their prescriptive nature can be overly conservative for research setups. Finally, flow-measurement standards such as ISO 9300 (critical-flow Venturi nozzles) offer superior accuracy and stability but at the cost of operating-range constraints, whereas ISO 5167 devices provide broader applicability with modestly higher uncertainty. Collectively, these standards differ in prescriptiveness, flexibility, and uncertainty-analysis depth. Understanding these trade-offs helps practitioners select the most appropriate framework depending on whether the test campaign is contractual, developmental, or research-focused.

## 3. Inlet and Ducts

Instrumentation of inlets and inter-ducts quantifies flow quality delivered to downstream components. It helps determine pressure recovery, total pressure and temperature uniformity, distortion, swirl, boundary-layer state, and turbulence. The results feed the compressor operability margins and map repeatability. In general, two measurement planes are recommended, far-field reference—which is upstream of contraction—or Bellmouth for total ambient conditions and the delivery plane at the aerodynamic interface plane (AIP) or just upstream of the downstream component. Additional planes like the S-ducts are used for diagnosing curvature or secondary flows.

### 3.1. Typical Instrumentation Suit

The typical instrumentation suit is composed out of the following:

- Total and static pressure measurements: Kiel probes which are insensitive to yaw, Pitot-static tubes, multi-hole (3–7 hole) probes, and rakes for circumferential and radial surveys.
- Temperature measurements: fast-response, shielded thermocouples, or thin-film RTDs and other total temperature probes with recovery correction.
- Flow angle and swirl measurements: calibrated multi-hole probes and cobra probes in low-speed ducts.
- Boundary layer and turbulence measurements: Pitot traverses, Preston tubes, hot-wire or hot-film anemometry within the proper temperature limits and surface hot-film arrays.
- Unsteadiness measurements: high-frequency pressure transducers (flush-mount) for buffet and/or pulsation, dynamic temperature (via fine thermocouples) if necessary.
- Optical measurements (as the assembly allows): PIV or LDV windows for research rigs, schlieren, or BOS for large scale gradients.
- Surface measurements: static taps, wall shear oil-film interferometry if there is optical access, temperature paints for icing or other heat-transfer studies.

### 3.2. Core Metrics

To ensure consistency in evaluating inlet and duct performance, a set of core metrics and standardized definitions is applied, covering pressure recovery, distortion, swirl, turbulence intensity, and boundary-layer characteristics.

Total pressure recovery is defined as the ratio of the average total pressure at the aerodynamic interface plane (AIP) to the free-stream or reference total pressure upstream of the inlet (Equation (1)). It quantifies the ability of the inlet to deliver flow with minimal total pressure loss due to friction, separation, or shock interactions. A recovery value close to unity indicates low loss, whereas lower values reflect higher energy dissipation in the duct.

(1) $$\eta_{P0}=\frac{\bar{P_{0,AIP}}}{P_{0,ref}}$$

where $\eta_{P0}$ is the total pressure recovery coefficient, $\bar{P_{0,AIP}}$ is the area-averaged total pressure measured at the aerodynamic interface plane (AIP), $P_{0,ref}$ is the free-stream or upstream reference total pressure, usually measured ahead of the inlet or at ambient conditions.

The area-averaged total pressure is obtained across the AIP by using Equation (2).

(2) $$\bar{P_{0,AIP}}=\frac{\int_{A_{AIP}}P_{0}\left(x,y\right)U_{n}(x,y)dA}{\int_{A_{AIP}}U_{n}(x,y)dA}$$

where $P_{0}\left(x,y\right)$ is the local total pressure at a point on the AIP, $U_{n}(x,y)$ is the local flow velocity component normal to the AIP, dA is the elemental area on the AIP, and $A_{AIP}$ is the total area of the aerodynamic interface plane.

This mass-flux-weighted average ensures that high-velocity regions contribute proportionally more to the mean value than low-velocity regions, making it representative of the effective flow delivered downstream.

However, in practice, the simplified discrete form of Equation (2) is applied, according to the usage of rakes and sectorized AIP surveys. This practical form is presented in Equation (3).

(3) $$\bar{P_{0,AIP}}=\frac{\sum_{i=1}^{N}P_{0,i}w_{i}}{\sum_{i=1}^{N}w_{i}},\;\;\;\;w_{i}=\rho_{i}U_{n.i}A_{i}$$

where $P_{0,i}$ is the total pressure measured at port or sector i, $w_{i}$ is the mass-flux weight for i, $\rho_{i}$ is the local density, $U_{n.i}$ is the local component normal to the AIP from multi-hole probe or derived from $P_{0,i}$, $p_{i}$, and $A_{i}$ is the area represented by the port or sector I, either sector ring area, or Voronoi cell.

Several simplifications are usually made, for example: (a) if the density variations are small, $\rho_{i}$ can be considered constant, so $w_{i}\propto U_{n,i}A_{i}$; (b) if only pressure data is available, estimate $U_{n,i}$ from $P_{0,i}$, and $p_{i}$ using the appropriate compressible relation for the test Mach number; (c) if the sectoring is uniform, with equal $A_{i}$ and negligible $U_{n}$ variation, the average reduces to a simple arithmetic mean of $P_{0,i}$.

The equation describing the circumferential total-pressure distribution of an assumed sector θ is presented in Equation (4).

(4) $${DC}_{\theta }=\frac{\bar{P_{0,AIP}}-min(P_{0,\;\theta })}{\bar{P_{0,AIP}}}$$

where ${DC}_{\theta }$ represents the circumferential distortion coefficient for a chosen angular sector θ and $min(P_{0,\theta })$ is the lowest sector-averaged total pressure in the selected circumferential band θ.

This metric quantifies the worst localized pressure deficit relative to the mean total pressure across the AIP. A larger ${DC}_{\theta }$ indicates more severe non-uniformity which can drive compressor instabilities or stall. The sector angle is chosen to reflect the engine’s circumferential sensitivity. Usually, for military and civil aeroengines a standardized θ=60° is used. In practice, the discreate version of the circumferential distortion metric is applied with rake or sector measurements. Here, the AIP is divided into circumferential sectors (60° arcs). Within each sector, pressures from rake probes are averaged, often with area or mass-flux weighting. The sector with lowest mean pressure is identified. The difference between this sector and the overall AIP average, normalized by the average, gives the distortion coefficient.

Circumferential distortion (${DC}_{\theta }$) is particularly critical for compressor operability, as non-uniformities around the annulus can trigger rotating stall or surge. In contrast, radial distortion (${DR}_{r}$), presented in Equation (5), primarily affects blade loading distribution across the span, influencing efficiency, secondary flows, and local stall inception near hub or tip regions. In the case of radial distortion, the AIP is divided into concentric rings (hub to casing) and the probe data within each ring are averaged (either area or mass-flux weighted). The scope is to identify the ring with the lowest total pressure mean. The deficit between this ring and the overall AIP average, normalized by the average, defines the radial distortion coefficient.

(5) $${DR}_{r}=\frac{\bar{P_{0,AIP}}-min(\bar{P_{0,\;k}})}{\bar{P_{0,AIP}}}$$

where ${DR}_{r}$ represents the radial distortion coefficient for ring r, $min(\bar{P_{0,k}})$ is the averaged total pressure within concentric ring k of the AIP, and the $min(\bar{P_{0,k}})$ is the lowest of the ring averages across all radial bands.

The swirl angle measurements are conducted via multi-hole probes which are calibrated in calibration tunnels to relate port pressure distributions to local flow vector components. This calibration is based on the cylindrical coordinates of the local velocity components, where $W_{x}$ is the axial velocity component aligned with the duct axis, $W_{\theta }$ is the tangential or circumferential velocity component, and $W_{r}$ is the radial velocity component. The local flow angles are described by Equations (6) and (7).

(6) $$\alpha ={tan}^{-1}\left(\frac{W_{\theta }}{W_{x}}\right)$$

(7) $$\beta ={tan}^{-1}\left(\frac{W_{r}}{W_{x}}\right)$$

where α is the yaw angle or circumferential swirl angle and β is the pitch angle or radial flow angle. α quantifies the magnitude and direction of swirl in terms of circumferential deviation, relative to the axial flow, and β quantifies the radial flow deviation which is often linked to secondary flows or boundary-layer growth.

When reporting swirl measurements, both mean values (indicating the bulk swirl bias), and RMS fluctuations (quantifying the unsteadiness of the flow) should be provided. It is essential to clearly state the adopted sign convention (e.g., positive α defined as clockwise swirl when viewed downstream) and the coordinate system employed, as these conventions differ across ISO, SAE, and NASA standards.

According to SAE definitions, in addition to local yaw and pitch angles from multi-hole probes, inlet swirl is commonly quantified with non-dimensional descriptors: the swirl intensity (SI) and swirl directivity index (SDI). The SI expressed the overall tendency of the flow to swirl and represents a mass-flux-weighted average of the tangential-to-axial ratio across the AIP. Hence, the SI is a measure of the magnitude of swirl.

The SDI indicates whether swirl is predominantly one-sided (values near 1) or balanced in both directions (values near 0). SDI indicates the directional bias of swirl (e.g., strongly biased swirl can destabilize compressors more severely than alternating swirl).

Governing equations of SI and SDI can be found in Equation (8) and Equation (9), respectively.

(8) $$SI=\frac{1}{AIP}\int_{A_{AIP}}\frac{W_{\theta }}{W_{x}}dA$$

(9) $$SDI=\frac{\left|\int_{A_{AIP}}W_{\theta }dA\right|}{\int_{A_{AIP}}\left|W_{\theta }\right|dA}$$

Both metrics are typically reported alongside mean and RMS swirl angles at the AIP to provide a full picture of inlet flow distortion.

Turbulence intensity is a non-dimensional measure of velocity fluctuations relative to the mean flow, defined as:

(10) $$Tu=\frac{u^{\prime }}{U}\times 100\%$$

where Tu is the turbulence intensity, u′ is the root mean square (RMS) value of the velocity fluctuations, and U is the mean velocity (typically the axial component, $W_{x}$).

Low values, below 1%, indicate nearly laminar or very uniform flow, typical of low-turbulence calibration tunnels. Moderate values 1–5% are representative of engine inlets and ducts under normal operation, and higher values, above 5%, may occur downstream of boundary layers, flow separations, or screens and can strongly influence compressor stability and heath transfer.

Usually, u′ is obtained from hot-wire or hot-film anemometry, fast-response multi-hole probes, or optical methods (PIV/LDV) when optical access is available.

In this case proper temporal resolution is essential with sampling rates at least an order of magnitude above the dominant turbulence frequencies.

The boundary layer (BL) characterizes the region near the duct or inlet wall where viscous effects dominate. Its properties are typically described through several integral parameters obtained from the local velocity profile U(y).

Boundary layer thickness (δ) is the distance from the wall to the location where the local velocity reaches 99% of the free-stream velocity $U_{\infty }$ (Equation (10)).

(11) $$\delta :U(y=\delta )\approx 0.99U_{\infty }$$

The displacement thickness ($\delta^{*}$) is the effective reduction in the duct cross-sectional area due to the slowing of flow in the boundary layer:

(12) $$\delta^{*}=\int_{0}^{\delta }\left(1-\frac{U(y)}{U_{\infty }}\right)dy$$

The momentum thickness quantifies the momentum loss caused by the velocity deficit in the boundary layer (Equation (13)).

(13) $$\theta =\int_{0}^{\delta }\frac{U(y)}{U_{\infty }}\left(1-\frac{U(y)}{U_{\infty }}\right)dy$$

The shape factor (H) is described as the ratio of the displacement thickness to momentum thickness. H provides insight into the velocity profile shape, H≈1.3−1.4 (typical turbulent BL), H≈2.5 (typical of laminar boundary layer), and higher H >3−4 indicates separation tendency. Thus, H is widely used as a diagnostic metric for assessing boundary-layer state and the likelihood of adverse pressure-gradient separation in inlets and ducts.

### 3.3. Probe Layout and Traversing

The accuracy of inlet and duct flow characterization depends strongly on how probes are distributed across the AIP. A well-designed probe layout ensures adequate spatial resolution while minimizing measurement interference and flow blockage.

Several considerations should be taken into account while designing the probe layout, such as: (a) circumferential resolution, (b) radial resolution, (c) probe type selection, (d) possible blockage and interference, (e) traversing strategies and calibration and alignment.

These considerations are briefly explained below.

Circumferential resolution: the AIP should be subdivided into a sufficient number of circumferential sectors to resolve potential distortions. At least 12 sectors are typically recommended for ${DC}_{60}$ analysis, although a finer division (24 or more) may be required for inlets with strong secondary flows or complex S-duct geometries.

Radial resolution: Within each sector, a minimum of five radial measurement stations is suggested to capture boundary-layer growth and radial gradients: near-wall (≈2–5% span), inner boundary layer (~15% span), midspan (~50%), outer span (~85%), and near-hub or casing (within 2–5% of wall proximity). Additional points may be added in regions where steep gradients are expected (e.g., near hub-corner separations).

Probe type selection: Bellmouth or Kiel probes are preferred for total-pressure measurement in flows with yaw angles up to ±30°, as their design minimizes directional sensitivity; multi-hole probes (3-, 5-, or 7-hole) should be employed when swirl or curvature exceeds ±5–7°, allowing simultaneous determination of pressure and flow angles, while straight Pitot-static probes may be sufficient in quasi-uniform axial flows but are prone to yaw-induced errors.

Possible blockage and interference: total probe blockage should not exceed 1–2% of the duct area to avoid artificial distortion of the measured flow field. Rakes and traversing arms must be carefully designed with streamlined supports and staggered stem placement to reduce mutual wake interference. CFD assessments or empty-duct calibrations are often performed to quantify and correct blockage effects.

Traversing strategies: in research test rigs, traversing mechanisms allow probe sweeps across circumferential or radial directions, providing continuous data with higher resolution than fixed rakes. Stepwise traversing combined with synchronized data acquisition is commonly used to reconstruct complete contour maps of pressure, temperature, and swirl across the AIP.

Calibration and alignment: proper angular alignment of multi-hole probes with respect to the duct axis is essential; small misalignments can lead to significant errors in reconstructed swirl angles. Alignment jigs, laser tools, or pre-calibrated mounts are often employed to minimize this uncertainty.

A probe layout that balances spatial resolution with minimal disturbance is fundamental for obtaining reliable AIP surveys. Poorly designed probe distributions risk underestimating distortion, mischaracterizing swirl, or artificially altering the flow field through probe-induced blockage.

Temperature measurements must be corrected for the probe recovery factor *r* and for radiative effects when strong temperature gradients are present. The use of shielded junctions and low-conduction probe supports help reduce thermal bias. To ensure consistency, temperature data should be synchronized with pressure measurements so that total-to-static properties can be derived accurately.

### 3.4. Uncertainty and Calibration

Reliable inlet and duct measurements require careful calibration of probes and rigorous quantification of uncertainties. Multi-hole probes should be calibrated in a dedicated facility (often a low-turbulence wind tunnel) over the yaw and pitch ranges expected during inlet operation, as well as across representative Reynolds and Mach numbers. Calibration maps are then used to reconstruct velocity vectors and swirl angles from port pressure differentials. Kiel and Pitot probes must also be verified against a reference jet or tunnel to confirm their yaw-insensitivity and recovery coefficients. This verification should be repeated after high-temperature or extended campaigns, since probe geometry and thermal stresses may alter their calibration constants.

Uncertainty analysis should follow recognized methodologies such as ASME PTC 19.1.

The total expanded uncertainty should be reported with a clear coverage factor (commonly k = 2 for 95% confidence), along with the effective acquisition bandwidth to demonstrate that the instrumentation adequately resolves the relevant flow features. In inlet applications, where flow distortion and swirl can vary rapidly with operating condition, documenting these limits is critical to ensure the fidelity and repeatability of AIP surveys.

Table 1 summarizes a representative uncertainty budget for inlet and duct instrumentation. Typical contributors include calibration accuracy, probe alignment, thermal effects, and spatial resolution across the AIP. Expanded uncertainties (coverage factor k = 2) are reported for the principal measured and derived quantities, ranging from ±0.2% for pressure measurements to ±3–5% for velocity components and ±0.01–0.03 for distortion coefficients. Clear documentation of the applied methodology (ASME PTC 19.1), coverage factor, and acquisition bandwidth are essential to ensure traceability and comparability of AIP survey results.

## 4. Centrifugal Compressors Diffusers Instrumentation: Techniques and Principles

In practical terms, accurate measurement and monitoring of diffuser flow characteristics directly influence compressor operability, efficiency, and mechanical integrity. Even modest variations in diffuser performance, such as a 1–2% reduction in static pressure recovery or a localized total-pressure deficit of the order of 3–5% at the diffuser exit, can shift the surge line appreciably and reduce the stable operating margin of the stage. Quantitatively, rotating stall cells typically induce circumferential pressure modulations of 2–10% of mean diffuser static pressure, while deep-surge cycles generate low-frequency oscillations that may exceed 20–40% of the diffuser-to-volute pressure rise. Detecting these variations enables operators to identify early signs of flow breakdown, actively adjust variable-geometry or bleed systems, protect the impeller from cyclic loading, and maintain compressor efficiency under part-load or transient conditions. Thus, comprehensive diffuser flow control, supported by reliable measurements of pressure, temperature, flow angle, and unsteady behavior, is essential not only for research accuracy but for real-world applications such as aircraft ECS compressors, turbochargers, industrial blowers, and process-gas machinery, where unmonitored diffuser instabilities directly translate into performance loss, increased vibration, and reduced component lifetime.

### 4.1. Pressure Measurements for Diffusers

#### 4.1.1. Total Pressure

Total pressure measurements are performed usually on the inlet/discharge of a compressor using Kiel probes [30] or rakes for robust, yaw-tolerant stagnation pressure, common on multi-position rakes at the inlet and exit. The shroud of a Kiel probe houses a Pitot tube [31], functioning as a flow straightener, minimizing errors caused by variations in flow angle [32]. As a result, Kiel probes provide accurate stagnation pressure measurements without the need for aerodynamic calibration, but they too need calibration for recovery and yaw sensitivity.

In compressor diffusers, Kiel probes are deployed where flow is highly non-uniform and swirling: immediately at the impeller-diffuser interface, within vaned or vaneless diffuser passages (near throat and mid-span), and at the diffuser exit–collector entry to map pressure recovery and circumferential gradients. Rake configurations are typically circumferential annulus rakes (evenly spaced around the casing at the diffuser exit), sector arrays that resolve one wedge at high-resolution, and radial or spanwise rakes inserted through casing ports to capture hub-to-shroud distributions. Recent improvements target lower blockage and higher fidelity: additively manufactured rake bodies with thin-wall Inconel [33] or Ti Kiel heads [34] for tight tolerances, PEEK/ceramic thermal breaks to reduce conduction bias [35,36], miniaturized piezoresistive sensors integrated inside the shroud for kHz-rate unsteady measurements [37], optional purge ports to mitigate fouling [38], and quick-disconnect manifolds with internal routing that preserve symmetry while speeding setup and calibration [39].

**Figure 1:**
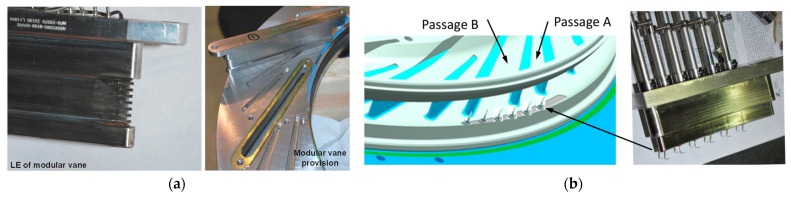
Diffuser model: (**a**) model depiction of diffuser exit measurement, (**b**) actual hardware [40].

**Figure 2:**
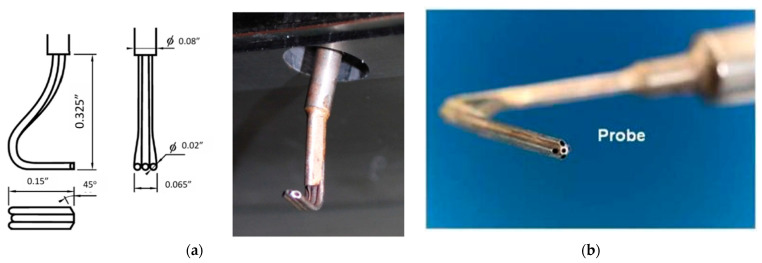
Multi-hole probes for pressure measurements: (**a**) 3-hole [40]; (**b**) 5-hole [43].

Examples of Kiel probes rakes can be observed in Figure 1 and Figure 2.

Alternatives to Kiel heads for stagnation pressures include cone Pitot probes (conical tip) [41], rounded/hemispherical Pitots, and multi-hole probes such as the cobra or three-hole example in Figure 2a [42] and five-hole [43] probes (Figure 2b). Cone [44] and rounded Pitots [45] allow arrangement in sector rakes at the diffuser’s exit and better incidence tolerance than straight Prandtl tubes but require aerodynamic calibration and precise alignment. Multi-hole probes are especially used because they can measure the flow angles (alfa, beta), allowing angle correction of total pressure where direction varies strongly through the vaned or vaneless passages and into the collector. Fast-response miniature Pitots [46] with short pneumatic lines or embedded piezoresistive sensors provide kHz-rate dynamic total pressure in the case of unsteady diffuser studies, such as stall cells and surge precursors, at the cost of higher sensitivity to blockage and installation effects compared with Kiel rakes.

**Figure 3:**
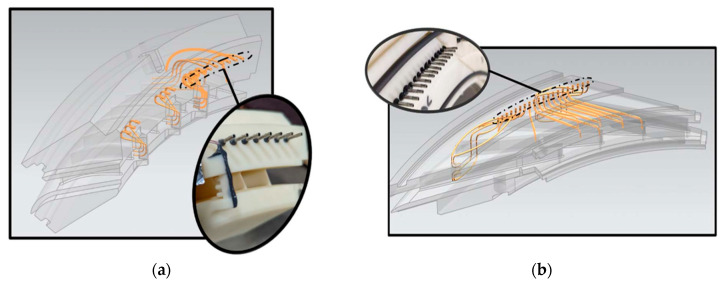
Diffuser instrumentation: (**a**) deswirl internal instrumentation channels and instrumentation locations, (**b**) internal instrumentation channels and egress design [40].

Figure 3 illustrates a possible diffuser instrumentation setup used to acquire flow measurements for validation and performance analysis. In Figure 3a, the internal instrumentation channels and probe locations are shown, including those positioned within the deswirl region to capture pressure and flow angle data in highly non-uniform areas. Figure 3b highlights the internal routing of instrumentation channels and the design of the egress paths through the casing. These allow probe access with minimal intrusion into the flow domain, while also maintaining structural integrity.

#### 4.1.2. Static Pressure Ports

General recommendations suggest using wall, burr-free, flush-mounted holes. Static pressure measurements provide the backbone for evaluating pressure recovery and loss coefficients and for diagnosing separation and circumferential non-uniformity.

The flush, burr-free wall taps are best if machined normal to the surface with small inner diameters (between 0.5 and 1.0 mm) and sharp entrances to minimize local disturbance. The taps are recommended to be distributed circumferentially and radially or spanwise (hub–mid–shroud), and on vaned diffusers, placed at throat, mid-passage, and exit to resolve the static rise through the passage. A dedicated static ring spool at the diffuser exit with many taps enables robust area-averaged $p_{s}$ and circumferential profiles while limiting blockage [47]. Transmission lines should be short, equal, and low-compliance, with matched internal volumes and nearby transducers. The measurement line is recommended to include purge/bleed points and to avoid water traps. Prior to testing, the line frequency response (step/pop test) should be tested and, if damping is required, amplitude/phase effects on unsteady content must be documented. For studies of rotating stall or surge, high-bandwidth piezoresistive wall sensors are to be co-located with taps to capture kHz-rate unsteadiness while the taps provide the DC level. Both plane-averaged and spatially resolved static pressures are reported and consistent reference conditions to form pressure recovery and loss metrics must be used [48]. Uncertainty is quantified from tap geometry and installation, line dynamics, sensor accuracy and calibration, and repeatability, since small biases in $p_{s}$ can materially affect inferred diffuser performance.

### 4.2. Pressure and Temperature Sensitive Paints

Recent work offers several promising upgrades for static-pressure measurements that translate well to compressor diffusers. At NASA Ames, multi-camera unsteady pressure-sensitive paint (uPSP) systems and refined acquisition workflows have matured to deliver high-density pressure maps on internal surfaces, a capability that can be ported to diffuser walls for steady or kHz-rate surveys with temperature correction [49].

Lifetime-based PSP has also advanced: on-chip accumulation with continuous-motion capture simplifies synchronization with conventional force/pressure data, easing diffuser campaigns where access is limited [50]. Time-resolved PSP has been demonstrated in complex internal compressible flows (a 3D inlet/isolator), indicating that the same paint/illumination/calibration methodology can map diffuser passages under swirl and separation [51].

For probe-based methods, a dynamic zonal KD-tree calibration for five-hole probes reduces large-angle “inner-boundary” errors, improving static-pressure accuracy in strongly yawed fields typical of diffuser exits [52]. Test infrastructure has likewise evolved: a 2025 stationary-component rig integrates dense wall static taps with multi-hole/total probes, providing a modern template for high-resolution diffuser mapping without rotor blockage constraints [53].

Complementary unsteady wall-pressure arrays—flush piezoresistive sensors with spectral analysis—have been added to open compressor test cases and can be applied on diffuser/collector walls to capture rotating-stall precursors. Finally, emerging conformal pressure sensor arrays (iontronic/MEMS) show sub-100 Pa-class resolution with ultrathin form factors; while demonstrated on aerodynamic surfaces, their conformality makes them attractive for instrumenting diffuser walls where conventional taps are intrusive [54].

For diffuser studies, temperature sensitive paints (TSP) provide global, high-density surface temperature fields on vanes and end-walls that can be converted into local heat-transfer coefficients under known thermal boundary conditions [55]. TSP uses a luminophore embedded in a thin binder; its emission intensity (or lifetime) varies with temperature, so a pre-test calibration is required (painted coupon or in situ two-point reference). Practical setups use tiled coatings for access, LED illumination with band-pass imaging, and a few thin-junction TC/RTD references to anchor absolute temperature and track drift. Compared with PSP, the effective bandwidth is typically limited by the thermal time constant of the paint-substrate system; lifetime-based TSP formulations and low-thermal-mass substrates can extend response for transient mapping. Key error sources—non-uniform illumination, photodegradation, coating thickness/aging, and emissivity changes—are mitigated with flat-field correction, frequent references, and consistent surface prep. In turbomachinery, combined PSP/TSP has been demonstrated on compressor hardware and remains attractive in diffusers, where dual-luminophore or two-color strategies help decouple pressure and temperature on painted surfaces [56].

### 4.3. Temperature Measurements

Total temperature measurements. In compressor rigs, accurate T0 at the inlet, impeller exit, and diffuser/collector entry is essential for enthalpy rise, power, and efficiency. Labs typically use thin-junction thermocouples (Type K, Class 1 [57]) or thin-film RTDs [58] packaged as shrouded (“Kiel/TAT-style”) total-temperature probes to improve recovery in yawed, swirling flow. *T*0 rakes should be co-located with total-pressure rakes in the same plane to avoid sampling mismatch, and a recovery-factor correction—validated by bench or in situ calibration—must be applied since probe recovery is rarely unity and depends on Mach and Reynolds numbers. The dominant biases are velocity (recovery) error, conduction through stems and mounts [33], and radiation from hot casings. Low-mass junctions should be mitigated via thermal breaks (ceramic/PEEK spacers [59]), short stings, and radiant shielding/view-factor control. For transient work (such as stall or surge), miniature or thin-film [60] elements with short leads for kHz-class response can be used and synchronized with unsteady wall-pressure sensors. Both plane-averaged and spatially resolved *T*0 must be reported and included in the uncertainty budget instrument tolerance and calibration (Type B), in the dwell-based repeatability (Type A), in the recovery-factor uncertainty, and in any radiation and conduction corrections because small errors in ΔT0 translate directly into large changes in derived efficiency.

**Figure 4:**
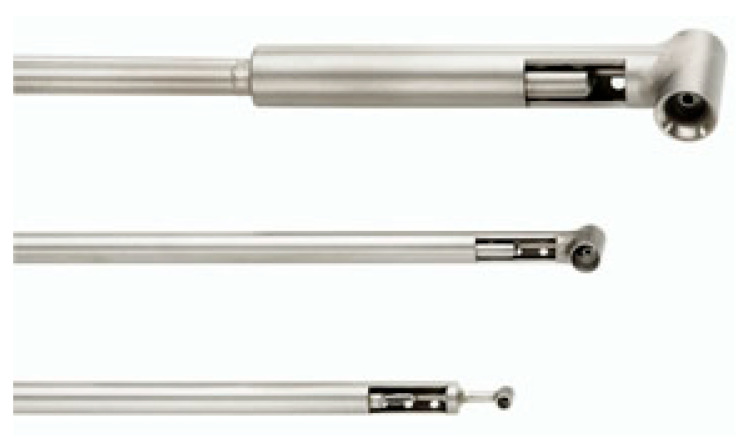
Combination pressure-temperature probe [61].

Figure 4 presents a temperature-pressure Kiel-TAT probe [61].

### 4.4. Flow Visualization and Velocity Measurement

#### 4.4.1. Mass Flow Rate Measurements

In compressor rigs, the total mass flow used to normalize diffuser maps is almost never measured in the diffuser; instead, it is measured in a well-conditioned straight run of the loop using critical-flow Venturi nozzles (CFVNs) per ISO 9300 [20], which, once choked, renders the indicated mass flow largely insensitive to downstream disturbances, which is ideal when the diffuser or collector introduces unsteadiness. Properly calibrated CFVN manifolds have demonstrated expanded (k ≈ 2) uncertainties down to ~0.07–0.09% on air, providing a robust reference for diffuser performance normalization [62].

When sonic nozzles are impractical (gas mix, pressure ratio, turndown), facilities employ differential-pressure primary elements per ISO 5167-1:2022 [21] and design the pressure signal transmission lines per ISO 2186 [25] so that dynamics (lag/attenuation) do not corrupt measurements near surge.

If a plane-specific flow through (or just downstream of) the diffuser must be established, a velocity-area integration using traverses in a short straight spool can be performed following ISO 3966 [63] methodology (Pitot/Pitot-static with angle correction and full uncertainty propagation). For closed-loop or non-air rigs, Coriolis meters [64] are attractive as facility meters (direct mass flow and density), provided single-phase operation and pulsation limits are respected; contemporary guidance and reviews cover their use for gases and high-accuracy application.

Finally, when documenting diffuser campaigns, both raw mass flow rate and corrected mass flow rate are reported, and the primary-element model/calibration, meter-mounted *p*, *T* sensors, gas-property model, installation effects, and repeatability are included in the uncertainty budget. The templates from NIST CFVN represent a good benchmark for procedure and uncertainty statements [65].

#### 4.4.2. Velocity Measurements

**Figure 5:**
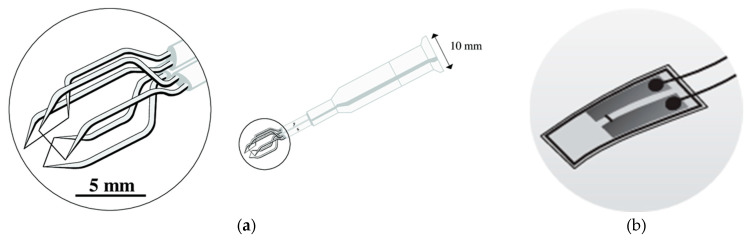
(**a**) Triple HW configuration [66], (**b**) thin-film (glue-on probe) [66].

Measuring velocity in the diffuser is not strictly required for contractual performance tests (pressures, *T*0, and mass flow rate are sufficient), but state-of-the-art programs now add velocity and unsteady measurements to explain losses, swirl decay, and instability. Modern setups pair dense static-pressure mapping—via taps/rakes and kHz-rate unsteady PSP on walls—with at least one mean-velocity/flow-angle traverse at the diffuser exit using multi-hole (cobra/five-hole) probes; recent large-angle calibrations improve accuracy in the strongly yawed outflow from the impeller. Where turbulence levels, spectra, and surge precursors matter, labs add hot-wire anemometry (CTA) in the core stream (X-/triple-wire with local ρ,T compensation as seen in Figure 5a [66]) and Figure 5b [67] hot-film (thin-film) sensors on hub/shroud or vanes to detect separation/reattachment and track wall shear dynamics. These datasets are synchronized with flush piezoresistive wall-pressure arrays and co-located *T*0/*Pt* rakes to close the energy and loss balance. Test stands increasingly use stationary-component rigs and additively manufactured diffusion systems to embed ports with low blockage and repeatable layouts, while facility-level critical-flow Venturi banks anchor mass flow accuracy for normalization.

### 4.5. Vibration and Acoustic Monitoring

The diffuser, being the component responsible for decelerating the high-velocity flow from the impeller and converting kinetic energy into pressure, is particularly sensitive to both mechanical and aerodynamic disturbances. Excessive vibrations or abnormal acoustic signals in the diffuser can indicate issues such as rotor–stator misalignment, blade fatigue, flow separation, or even early signs of stall and surge. Therefore, continuous monitoring enables early detection of potential problems, preventing catastrophic failures and reducing maintenance costs.

Vibration monitoring primarily focuses on mechanical disturbances that can affect the structural integrity of the diffuser and surrounding components. Accelerometers, typically piezoelectric or MEMS-based, are mounted on the diffuser casing or supporting structure to measure displacement, velocity, or acceleration. These sensors allow the capture of characteristic vibration signatures, including blade passing frequencies (BPF) and broadband mechanical noise. The collected signals are analyzed in both the time and frequency domains using tools like Fast Fourier Transform (FFT) or order tracking methods, which are particularly useful for variable-speed operations [67]. Advanced techniques, such as operational deflection shape (ODS) analysis and laser Doppler vibrometry [68], can visualize vibration patterns and identify localized structural responses, helping engineers pinpoint the sources of vibration.

Acoustic monitoring complements vibration measurements by detecting aerodynamic disturbances and pressure pulsations generated within the diffuser. Microphones or hydrophones can capture airborne or fluid-borne noise, while flush-mounted pressure transducers measure wall-pressure fluctuations directly [69]. Acoustic signals are typically analyzed through spectral analysis to identify tonal components, such as rotor–stator interaction frequencies and broadband noise indicative of flow separation or turbulence. Techniques like sound intensity mapping and acoustic beamforming can localize the sources of noise, providing further insight into diffuser performance and potential flow instabilities.

In practice, effective monitoring of centrifugal compressor diffusers often combines multiple instrumentation types to capture a comprehensive picture of the system’s health. Commonly used instruments include piezoelectric accelerometers, MEMS vibration sensors, flush-mounted pressure transducers, high-fidelity microphones, laser Doppler vibrometers, and data acquisition systems capable of high-frequency sampling. These instruments, integrated with advanced signal processing methods, enable engineers to correlate vibration and acoustic patterns with operating conditions, facilitating predictive maintenance and optimization of diffuser performance. By implementing such monitoring strategies, operators can enhance the safety, reliability, and efficiency of centrifugal compressors while minimizing unplanned downtime.

Based on the monitoring approach, each category—vibration or acoustic—requires specific instrumentation.

1. Vibration sensors—measure mechanical vibrations to detect imbalance, misalignment, or structural issues. In this category there are:

- Piezoelectric accelerometers: mounted on the diffuser casing, near the diffuser vanes, and on supporting structures to measure vibrations in multiple directions.
- MEMS accelerometers: small, lightweight sensors that can be mounted directly on diffuser walls or rotor housings.
- Laser Doppler Vibrometer (LDV): non-contact measurement for lab studies, aimed at diffuser blades or casing to capture vibrations without physical attachment.

Sensors should be mounted rigidly to avoid damping effects and positioned close to regions of high mechanical stress, such as blade roots, diffuser hub, or casing near rotor–stator interactions.

2. Acoustic sensors—capture aerodynamic noise and pressure pulsations caused by flow instabilities.

- Microphones: positioned outside the diffuser casing to capture airborne noise or in anechoic sections of test rigs.
- Hydrophones: if the diffuser handles fluid noise (like in water or liquid-gas testing), placed in flow channels.
- Pressure transducers/flush-mounted sensors: flush-mounted with the diffuser wall at selected locations along the hub, shroud, and near the diffuser vanes to measure wall-pressure fluctuations directly.

To identify aerodynamic disturbances, sensors are often positioned at critical points where flow separation, recirculation, or shock interactions are expected, such as leading edges of vanes, hub-shroud junctions, and diffuser throat or exit.

3. Data acquisition and signal processing

- DAQ Systems: high-speed acquisition units connected to accelerometers, microphones, and pressure transducers.
- Software tools: FFT analyzers, order tracking software, and operational deflection shape (ODS) analysis tools to interpret vibrations and acoustic patterns.

4. Optional advanced instrumentation

- Acoustic beamforming arrays: a set of microphones placed around the diffuser to localize noise sources.
- High-speed cameras or schlieren systems: for visualizing flow instabilities, often paired with acoustic measurements in laboratory setups.

Table 2 table outlines common sensor types used in diffuser diagnostics and their typical placement within the system.

Despite the broad range of available instrumentation, several technologies commonly used in diffuser research remain constrained by intrinsic limitations that restrict their applicability in modern high-speed compressor environments. Traditional intrusive probes such as Pitot tubes, Kiel heads, and multi-hole probes, continue to form the backbone of diffuser measurements, yet they are increasingly inadequate for detailed characterization of highly three-dimensional, transient flows. Their shortcomings include blockage sensitivity, restricted bandwidth, calibration drift at high temperatures, and susceptibility to alignment errors, making them unsuitable for resolving fast stall precursors or small-scale turbulence. Likewise, hot-wire and hot-film anemometry, once indispensable for turbulence studies, are now limited by fragility, temperature constraints, and position sensitivity, and thus rarely survive in harsh industrial compressor settings. Optical methods, although offering unparalleled spatial resolution, remain hindered by line-of-sight requirements, seeding challenges, mirror fouling, and the general impracticality of integrating optical windows into production machines. Meanwhile, key unresolved problems persist: bandwidth limitations in pressure/temperature probes for fully capturing surge-cycle dynamics; difficulties in synchronizing heterogeneous sensor types; lack of in situ calibration techniques for rotating environments; and insufficient robustness of emerging MEMS and conformal sensors for long-duration industrial operation. These gaps illustrate that, despite recent progress, diffuser instrumentation still lacks a universally applicable high-bandwidth, minimally intrusive sensing technology capable of real-time monitoring across all operating conditions. Table 3 syntheses the main shortcomings of the diffuser measurement techniques.

Comprehensive diagnostic systems for centrifugal compressors are increasingly designed as integrated platforms that combine pressure, temperature, vibration, acoustic, and, where available, optical measurements into a unified monitoring framework. Their purpose is to capture the full operational state of the compressor stage, from steady performance parameters to fast unsteady flow phenomena. The primary goal of such systems is early detection of abnormal behavior, surge precursors, rotating stall modes, diffuser flow separation, or structural vibration patterns, so that corrective actions can be initiated before performance degradation or mechanical damage occurs. By fusing multiple sensor types, these systems enhance diagnostic capability: high-bandwidth pressure transducers resolve instability signatures, thermocouples track thermal loading, accelerometers capture rotor-stator mechanical interactions, and data-driven models can infer unmeasured flow quantities. The advantages include higher reliability, robustness to noise, and improved situational awareness compared to single-sensor approaches. The beneficial effects are substantial: increased surge margin, reduced unplanned downtime, extended component life, and more efficient operation through adaptive control strategies. Such integrated diagnostic architectures, therefore, represent a critical evolutionary step toward resilient, intelligent compressor systems suitable for both industrial and aerospace applications.

## 5. Case Studies and Real-World Applications

Furthermore, several case studies from the literature are presented, detailing the instrumentation setups and experimental procedures used. The analysis emphasizes how these configurations affect the accuracy, sensitivity, and interpretation of the monitoring results.

### 5.1. Industrial/Retrofitted

#### 5.1.1. Pressure-Based Monitoring

**A.**  **DLR–Liebherr Single-Stage Centrifugal Compressor Facility**

The single-stage centrifugal compressor (SSCC) investigated at the German Aerospace Center (DLR) features an impeller with 15 unshrouded backswept blades, a vaneless diffuser, and an asymmetric volute. Originally designed by Liebherr Aerospace Toulouse SAS for aircraft cabin air-conditioning systems, the geometry was adapted by DLR to accommodate extensive instrumentation for detailed aeroacoustic and unsteady flow investigations.

**Instrumentation Setup—**To capture the unsteady flow behavior across the compressor stage, 19 miniature unsteady pressure transducers (Kulite XCE-062 (Kulite Semiconductor Products, Inc., Leonia, NJ, USA)) were flush-mounted in the impeller casing. These sensors were distributed asymmetrically at three meridional positions to prevent modal bias in the detection of stall precursors and rotating instabilities.

Complementary planar 2D-2C high-speed PIV measurements were performed upstream and downstream of the impeller (at 3.0r_1_ and 1.1r_2_). The PIV system employed dual-pulse diode-pumped solid-state lasers (Innolas Photonics Nanio Air 532-10-V-SP (Thorlabs Inc., Newton, NJ, USA)) providing up to 10 W average power at 26 kHz repetition rate, with pulse separations between 1 and 20 µs. The laser sheet thickness ranged from 250 to 450 µm, and tracer particles (<1 µm) were generated by a Vicount smoke generator. Image acquisition was conducted using a Phantom v1840 high-speed CMOS camera (Vision Research, Inc., Wayne, NJ, USA) at 54 kHz, with optical access provided by window-mirror systems tailored to each location.

**Figure 6:**
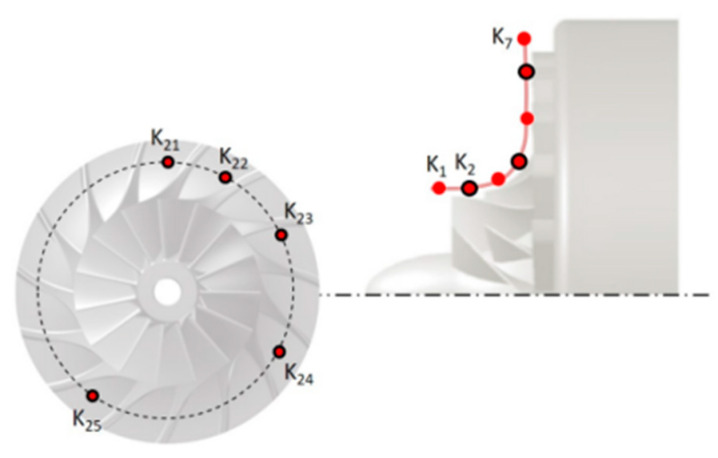
Positioning of the unsteady pressure probes [70].

**Figure 7:**
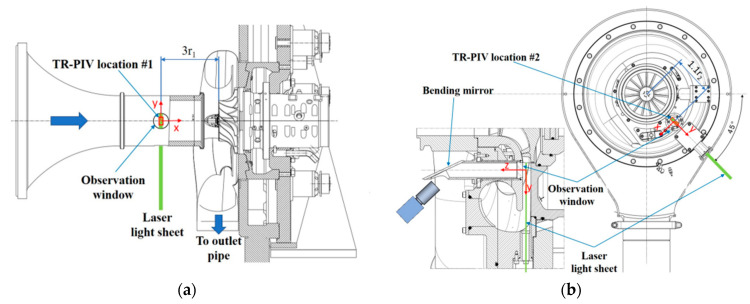
Instrumentation of DLR–Liebherr Single-Stage Centrifugal Compressor, measurement stations for TR-PIV: (**a**) inlet configuration; (**b**) impeller exit configuration [70].

Figure 6 presents the positions of the measurement probes and Figure 7 presents the measurements stations for TR-PIV.

**Data Acquisition and Experimental Procedure—**The pressure transducers, capable of resolving fluctuations as low as 0.07–0.12 mbar, were connected to a Dewetron 808 data acquisition system equipped with 24-bit A/D converters, operating at a sampling rate of 200 kHz, and a bandwidth of 150 kHz. Additional inputs included spindle speed, PIV triggers, microphone signals, and gap sensor data. Synchronization between multiple transient recordings was achieved via a satellite IRIG-B time reference, ensuring precise temporal alignment across instruments.

Advanced PIV processing (PIVview 3.9 and in-house Python tools) enabled interrogation window sizes down to 16 × 16 pixels (≈190 µm) at the inlet and 32–64 × 32 pixels in the diffuser, maintaining > 95% vector validation rates even under high turbulence conditions. A key strength of this setup is the synchronization of TR-PIV with the unsteady pressure measurements, allowing direct correlation of spatial velocity fields with pressure fluctuations in the casing. Over three terabytes of image data were collected, with each test point comprising multiple bursts of over 10,000 image pairs.

**Figure 8:**
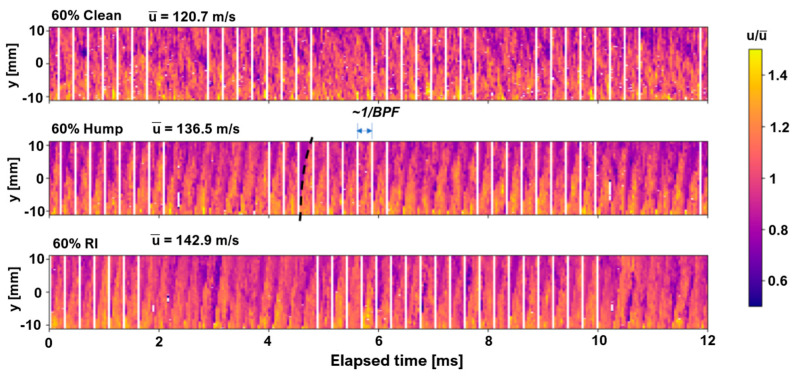
PIV time traces of circumferential velocity *u* of the impeller exit flow and along the radial direction *y* recorded with 26 kHz at location #2 near the casing wall at ‘clean’ (**top**), ‘hump’ (**middle**), and ‘RI’ (**bottom**) conditions [70].

**Influence on Results—**PIV time traces of circumferential velocity at the impeller exit flow are presented in Figure 8. The white bars represent measurements affected by laser reflections on the impeller blades.

High-speed, cross-synchronized measurements of velocity and unsteady pressure enabled detailed identification of rotating instabilities and mode-locked behavior near surge conditions. Spectral and coherence analyses revealed strong coupling between low-frequency velocity fluctuations and unsteady pressures, particularly upstream of the rotor, highlighting propagating disturbances that preceded flow instabilities. These results demonstrate the effectiveness of combined pressure-velocity diagnostics for understanding the onset and development of instabilities in centrifugal compressors.

**B.**  **Industrial Centrifugal Compressor Test Rig for Dynamic Pressure Measurements**

This investigation focuses on an industrial single-stage centrifugal compressor with a vaneless diffuser, which is representative of units used in sewage disposal systems. The compressor is driven by an 180 kW AC motor operating at 18,300 rpm, achieving a maximum pressure ratio of 1.88 at the design flow rate. The experimental facility was specifically configured to study the mechanisms of flow instability, stall, and surge using a dense network of high-precision dynamic pressure transducers.

**Instrumentation Setup—**Static and total pressure measurements were acquired via Pitot tubes at the compressor inlet and outlet, with a relative uncertainty below 0.26%, while thermocouples (±0.25%) monitored the corresponding total temperatures. Flow rate regulation was performed through a throttle valve system monitored by a Venturi flowmeter (accuracy better than ±0.5%).

The core of the instrumentation involved a multi-phase dynamic pressure measurement system. Kulite XCE-093 piezoresistive transducers were flush-mounted at seven radial locations spanning the impeller and diffuser inlet regions, with multiple sensors distributed circumferentially at each radius. Each sensor, with a 2.4 mm probe diameter and 0–300 kPa range, provided an accuracy of 0.05% full scale.

The signals from all sensors were acquired synchronously using eight NI 9215 acquisition cards connected to a DAQ-9178 chassis (National Instruments, Austin, TX, USA), achieving a combined measurement uncertainty below 0.5%. To accurately capture pressure fluctuations associated with blade passing, the sampling frequency was set to 20 kHz per channel, ensuring the correct resolution of the blade passing frequency (BPF = 2.44–4.88 kHz).

**Figure 9:**
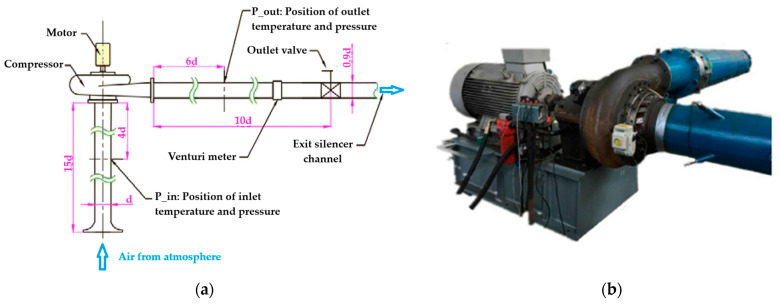
Test rig of the experimental compressor: (**a**) sketch of the test rig; (**b**) assembled test rig configuration [71].

**Figure 10:**
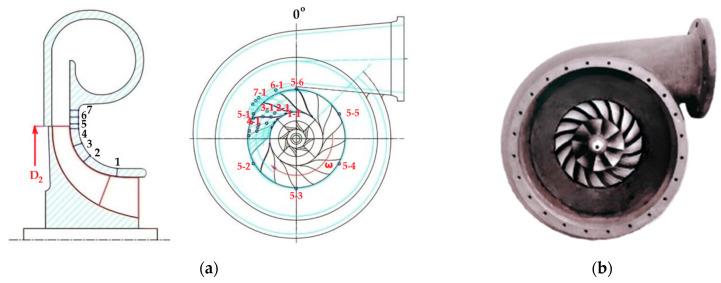
Multi-phase dynamic pressure test system: (**a**) distribution of dynamic pressure sensor transducers; (**b**) photograph of the experimental compressor [71].

The test rig of the experimental compressor is shown in Figure 9, and the arrangement of the dynamic pressure sensors is presented in Figure 10.

**Data Acquisition and Experimental Procedure—**During testing, the compressor was gradually throttled from the design point to deep surge. At each step, simultaneous time-resolved pressure signals from all transducers were recorded to capture the evolution of unsteady flow phenomena, including the onset and development of rotating stall cells and large-scale surge oscillations. These synchronized measurements formed the experimental foundation for analyzing the spatiotemporal characteristics of flow instabilities in the impeller–diffuser system.

**Influence on Results—**The dynamic pressure measurements enabled the identification of a three-lobe rotating stall mode propagating at approximately 30.8% of impeller rotational speed. The experimental data revealed that this mode originates in the diffuser region, intensifying as the flow rate decreases due to asymmetric pressure distribution at the impeller exit. The measurements further demonstrated how leakage flow near the mid-rear impeller region amplifies this instability, eventually leading to full surge.

The synchronized high-resolution pressure data also provided essential validation for unsteady numerical simulations and Dynamic Mode Decomposition (DMD) analysis, confirming that periodic pulsations near the impeller outlet generate alternating high- and low-pressure zones in the diffuser. These findings underscore the importance of distributed, high-frequency dynamic pressure instrumentation for accurately characterizing the precursors and the evolution of stall and surge phenomena in centrifugal compressors.

**C.**  **Dynamic Instrumentation for Surge Detection in a High-Speed Centrifugal Compressor Turbocharger**

This study investigates a high-speed centrifugal compressor turbocharger with a vaneless diffuser (max speed 185,000 rpm), focusing on establishing surge criteria applicable for real operational conditions. The experimental setup emphasizes high-frequency measurements of pressure, temperature, and acoustic signals to characterize the transient behavior of the compressor near surge.

**Instrumentation Setup—**The facility was instrumented with three types of dynamic probes:

- High-response pressure probes (Kulite) were installed at the impeller inlet (P1, 280° circumferential) and mid-diffuser section (P2, 50°). These probes have very high natural frequencies and can operate across a wide temperature range (−55 °C to 273 °C). The sampling frequency was set to 50 kHz, capturing small-scale instabilities and pressure perturbations up to the surge point.
- Fast-response thermocouples (Müller Instruments) were located near P1 and P2, with a minimum response time of 3 μs. Dynamic temperature measurements were obtained by summing the measured variation with the ambient temperature, providing reliable monitoring of thermal fluctuations in the flow.
- ICP microphones were positioned near the compressor shroud (S1) and the inlet duct (S2) to measure vibration-induced and aerodynamic noise, respectively. Microphone signals were sampled at 50 kHz; a low-pass filter (cut-off at 15% of shaft rotation frequency) was applied to separate mechanical vibration from aerodynamic features.

All sensors were connected to a DEWEsoft data acquisition system: pressure probes and thermocouples to DEWE-43 modules and microphones to SIRIUSi-ACC(+) modules, enabling synchronous acquisition of multiple signal types. Post-processing of the data was conducted within DEWEsoft.

**Figure 11:**
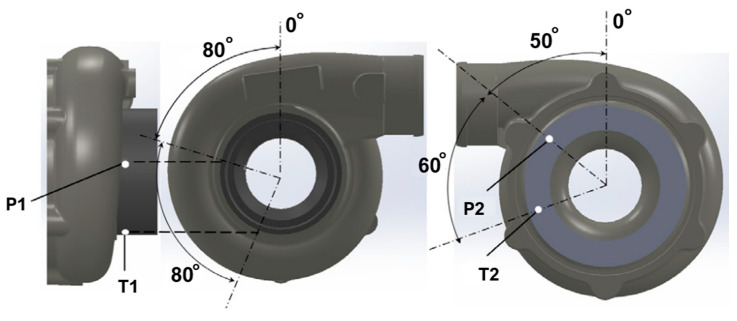
Location of pressure probes and thermocouples [72].

The location of the pressure probes and thermocouples is presented in Figure 11.

**Data Acquisition and Results—**The experiments captured surge behavior across three high-pressure-ratio centrifugal compressors. Analysis employed time-domain signals, variance maps, and power spectral density (PSD) plots. Key observations include:

- Dynamic pressure signals accurately reflected flow instabilities and small-scale perturbations in the impeller and diffuser, with the standard deviation (SD) slope increasing as mass flow approached surge.
- Microphone signals were less reliable for direct surge detection without filtering due to mechanical vibrations; post-processing was required to isolate aerodynamic contributions.
- Dynamic temperature measurements, particularly in the diffuser, provided the most effective indicator of surge onset. High-pass filtered SD maps displayed a clear rise-turn pattern at the minimum flow limit, across all rotational speeds and surge types.

**Influence on Results—**The high-frequency pressure and temperature probes enabled precise identification of surge points and characterization of the transient flow dynamics leading up to surge. Among the instruments, fast-response thermocouples were found to be the most effective for surge detection in engineering applications, while dynamic pressure measurements provided detailed insight into flow mechanisms at low flow rates, which is critical for understanding the onset of compressor instability.

**D.**  **Low-Specific-Speed Centrifugal Compressor Stage at the CSTAR Facility**

The experimental study was conducted at the Centrifugal Stage for Aerodynamic Research (CSTAR) facility, Maurice J. Zucrow Laboratories, Purdue University. Operational since 2015, the facility is designed for studying the aerodynamics of an axi-centrifugal compressor stage for aeroengine applications, with both steady and unsteady instrumentation to assess performance and flow structures. The CSTAR stage is a low-specific-speed centrifugal compressor operating at a design corrected speed of 22,500 RPM and a pressure ratio of approximately 3. Compressor performance was characterized by using temperature and pressure measurements along the flow path to capture both steady and transient behavior.

**Instrumentation Setup—**Temperature measurements were obtained using T-type thermocouples, while total temperature rakes were positioned at the impeller inlet, diffuser inlet, and turn-to-axial exit. Pressure measurements used Scanivalve Digital Sensor Arrays (DSA) to capture static and total pressures along the impeller, diffuser, and turn-to-axial, including spanwise and circumferential distributions. Optical access allowed for non-intrusive laser Doppler velocimetry (LDV) measurements of impeller exit flow structures.

**Figure 12:**
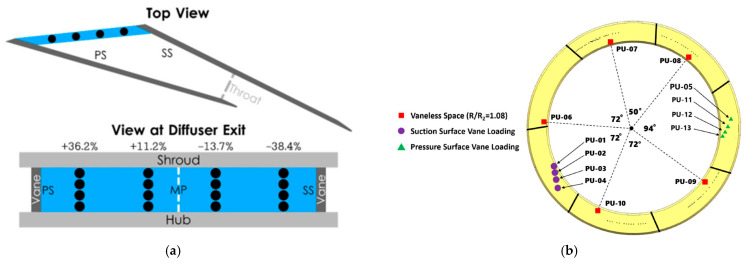
Position of the instrumentation setup: (**a**) position of diffuser exit rakes for baseline diffuser; (**b**) layout of Kulite transducers in CSTAR diffuser [73].

Figure 12 presents the instrumentation setup.

**Data Acquisition and Results—**The study examined diffuser inlet incidence, radial velocity, secondary flows, and their impact on diffuser performance. Key findings include:

- Incidence becomes more positive as mass flow decreases, with unsteady variations caused by impeller jets and wakes.
- Positive incidence improves diffuser effectiveness and reduces total pressure loss, while negative incidence reduces performance.
- Diffuser vane leading edges experience the highest loading and pressure differentials, particularly during surge, highlighting failure-prone regions.
- Spike-type stall originates in the vaneless space and can trigger full-stage surge with rapid flow reversal.
- Computational predictions using the BSL-EARSM turbulence model aligned reasonably with measurements, providing insight for safe operating limits.

**Influence on Results—**The results demonstrate that unsteady phenomena, rather than steady-state conditions, drive leading-edge diffuser vane failures. LDV measurements combined with computational models provide complementary insights into incidence, lift, and pressure variations, informing design and operational strategies to prevent mechanical failures.

#### 5.1.2. Acoustic and Vibration Instrumentation

**A.**  **Vibro-Acoustic Instrumentation of a Two-Stage Centrifugal Compressor for Fuel Cell Systems**

The investigated system is a high-speed, electric two-stage centrifugal compressor designed for fuel cell applications with output powers exceeding 100 kW. This compressor has been commercially implemented, making its experimental study directly relevant to industrial practice. The research aimed to characterize the compressor’s vibro-acoustic behavior during normal and surge operating conditions, providing a basis for surge prediction and control.

**Instrumentation Setup—**A comprehensive vibro-acoustic measurement system was employed to capture both pressure fluctuations and structural vibrations across the compressor stages. Sound pressure levels (SPL) were recorded using G.R.A.S. 40PH ½″ microphones (G.R.A.S. Sound & Vibration, Skovlytoften, Denmark), while vibration acceleration levels were measured using KISTLER triaxial accelerometers (Kistler Group, Winterthur, Switzerland). The measurement layout followed ISO 2151:2008 [74] guidelines, with microphones positioned at key locations—first-stage inlet, 45° from the first-stage outlet axis, second-stage inlet, 45° from the second-stage outlet axis, and second-stage outlet—each placed 1 m away from the compressor surface. The microphones covered a 20–20,000 Hz frequency range, while the accelerometers covered 20–10,000 Hz. Both systems sampled data at 48 kHz, ensuring sufficient temporal resolution for transient flow and vibration phenomena. Data acquisition and analysis were performed using the HEAD ArtemiS SUITE software.

**Figure 13:**
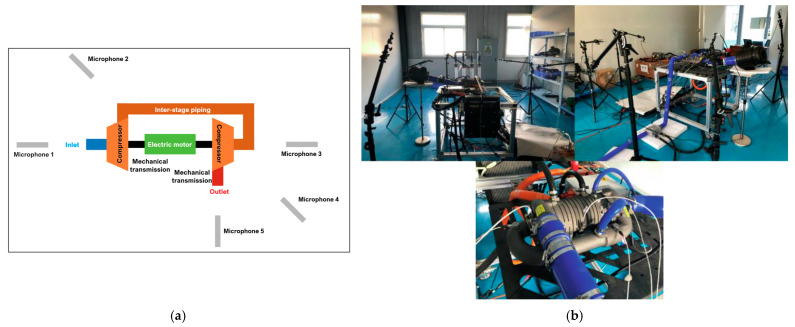
Layout of the compressor vibro-acoustic experiments; (**a**) the schematic diagram of the microphone arrangement, (**b**) the photographs of the experiments [75].

**Data Acquisition and Results—**The experimental campaign investigated the compressor behavior under near surge and deep-surge conditions. Using Short-Time Fourier Transform (STFT) analysis, transient acoustic signatures were visualized, enabling correlation between noise characteristics and flow instabilities. Results revealed that a rise in SPL within the 20–40% range of the rotor shaft frequency (RSF) serves as an early indicator of rotating stall, allowing prediction 1.5–3 s before onset. At deep-surge conditions, increased SPL and vibration amplitude were linked to strong outlet pressure pulsations. The layout of the vibro-acoustic experiments can be observed in Figure 13.

**Influence on Results—**The combined use of high-fidelity acoustic and vibration instrumentation provided a clear understanding of the dynamic coupling between aerodynamic instabilities and structural responses. The experimental data validated a mathematical surge model that accurately predicted surge frequencies across operating speeds with a maximum error of 1.95%. This validation underscores the crucial role of precise instrumentation in developing predictive models and designing effective surge control strategies for high-speed fuel cell compressors.

### 5.2. Research/Experimental Facilities

#### 5.2.1. Pressure-Based Instrumentation

**A.**  **Single-Stage Centrifugal Compressor (SSCC) Facility, Purdue University**

The Single-Stage Centrifugal Compressor (SSCC) facility at Purdue University is equipped with an experimental Honeywell centrifugal compressor designed for detailed investigation of diffuser and overall stage performance. The setup integrates a wide range of sensors for both steady-state characterization and unsteady flow diagnostics.

**Instrumentation Setup—**Steady-state performance is assessed using total pressure and total temperature probes positioned at key stations: compressor inlet (station 0), diffuser exit (station 5), and deswirl exit (station 6). A network of static pressure taps distributed along the flow path provides detailed information on the local pressure rise through the impeller, diffuser, and deswirl sections.

**Figure 14:**
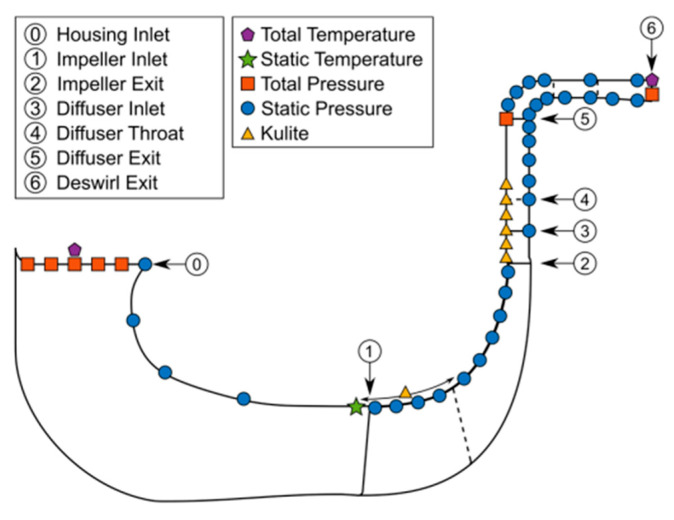
Cross-section of compressor and performance instrumentation [76].

To monitor flow recirculation at the impeller inlet, two thermocouples placed 180° apart upstream of the impeller leading edge capture circumferential temperature variations. In addition, fast-response pressure transducers are strategically installed throughout the compressor flow path. These sensors are critical for capturing high-frequency unsteady pressure signals associated with stall inception and surge phenomena. A cross-sectional representation of the compressor, showing the positions of the instrumentation, is provided in Figure 14.

**Influence on Results—**The use of fast-response transducers allowed detailed identification of distinct surge signatures as the impeller inlet flow transitioned from subsonic to supersonic conditions. Specifically, the data revealed that spike-type deep surge occurred under subsonic and supersonic conditions, while modal-type mild surge appeared near transonic inlet speeds. This high temporal resolution—made possible by the unsteady pressure instrumentation—enabled a clear differentiation between surge mechanisms and linked them to changes in inlet Mach number.

Moreover, combining steady-state pressure data with dynamic sensor signals facilitated a correlation between static pressure rise characteristics and instability onset, demonstrating the importance of distributed pressure instrumentation for diagnosing component-level contributions to overall stage behavior.

**B.**  **NASA Glenn Research Center—Centrifugal Compressor Diffuser Instrumentation Using DPIV and Pressure Transducers**

The NASA Glenn Research Center conducted advanced experimental investigations on a scaled Rolls-Royce Allison centrifugal compressor, originally designed for a flow rate of 1.66 kg/s and scaled up to 4.54 kg/s. The impeller and vaned diffuser were configured to achieve a pressure ratio of 4:1 at design mass flow, operating at a corrected design speed of 21,789 rpm with an exit tip speed of 492 m/s. The configuration included 15 main blades and 15 splitter blades with 50° backsweep, feeding a 22-vane diffuser.

**Instrumentation Setup—**The compressor was instrumented with a combination of Digital Particle Image Velocimetry (DPIV) and high-frequency dynamic pressure transducers, enabling simultaneous time-resolved pressure and velocity measurements in the non-stationary diffuser flow.

A compact light-sheet delivery system employing a periscope-type probe was inserted through the collector housing at a 90° bend downstream of the diffuser, allowing optical access to four diffuser vane passages. The illumination was provided by a pulsed laser light sheet, and the flow was globally seeded with alumina particles, ensuring high seeding density for robust image correlation.

The compressor casing was fitted with four fast-response Kulite pressure transducers (three rated at 340 kPa and one at 100 kPa). Transducers were placed at strategic circumferential and radial locations: two in the vaneless space between the impeller exit and diffuser inlet (at 343° and 35° from top-dead-center), one in the diffuser throat (at 43°), and one upstream of the impeller (25 mm ahead, at 338°). These sensors enabled real-time monitoring of pressure fluctuations and stall-cell propagation.

**Figure 15:**
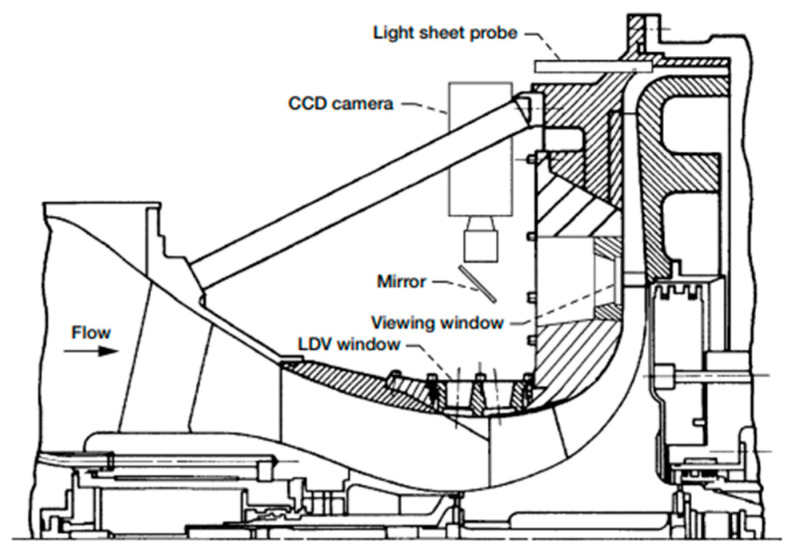
Cross-section of the centrifugal compressor facility [77].

**Figure 16:**
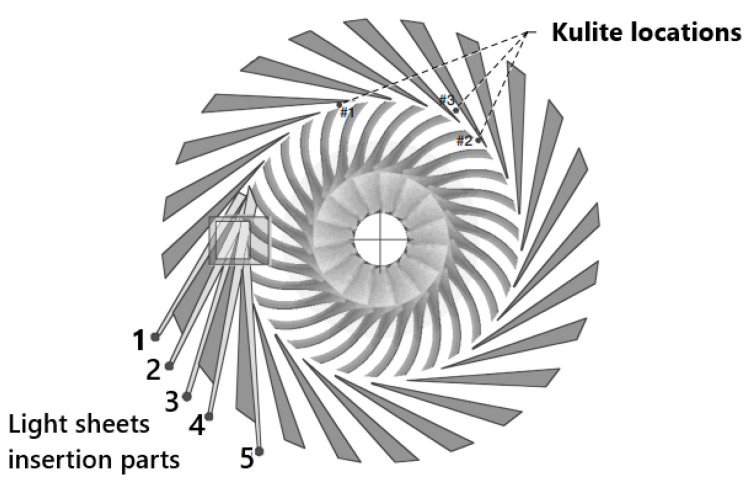
Schematic drawing of vaned diffuser and impeller showing the optical viewing port, light sheet probe insertion location, light sheet extension, and Kulite locations [77].

The cross-section of the centrifugal compressor facility is presented in Figure 15, with Figure 16 showing the positioning of the Kulite sensors.

**Influence on Results—**The combination of DPIV and dynamic pressure measurements provided unprecedented insight into the time evolution of flow unsteadiness during compressor surge and rotating stall. DPIV captured instantaneous velocity fields across multiple diffuser passages, revealing complex flow separation and reversal patterns during surge cycles, through the available optical port presented in Figure 16.

Meanwhile, the high-frequency Kulite transducers recorded stall precursors and rotating stall cells traveling at approximately 25–33% of the impeller rotational speed.

The synchronized use of optical and pressure instrumentation allowed researchers to correlate the pressure-wave evolution with local velocity field changes, offering the first time-resolved visualization of flow field breakdown during surge in a centrifugal compressor. Importantly, the measurements demonstrated that stall inception mechanisms are speed-dependent—initiating in the impeller region at low speeds (≤70%) and in the diffuser at design speed—thereby confirming the diagnostic value of combined dynamic and optical techniques for identifying instability origins.

**C.**  **Experimental Study of a Centrifugal Compressor Stage with Advanced Instrumentation at CSTAR**

**Figure 17:**
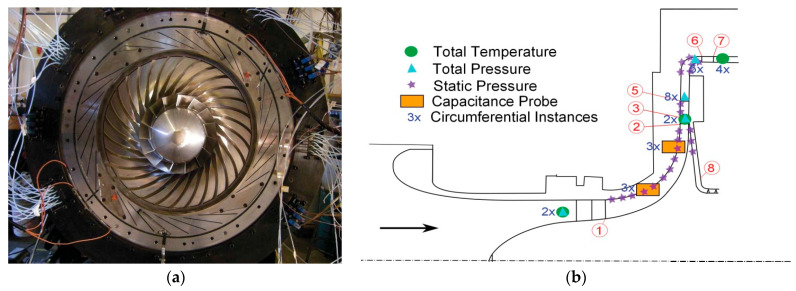
(**a**) CSTAR impeller and diffuser front view; (**b**) meridional view with instrumentation location [77].

The research was conducted at Purdue University’s Centrifugal Stage for Aerodynamic Research (CSTAR) facility. The tested centrifugal compressor stage has a design corrected speed Nc of 22,500 rpm and operates across engine-representative Mach numbers. The rear-mounted driveline consists of an ABB AC electric motor. Instrumentation locations along the flow path are summarized in Figure 17.

**Instrumentation Setup—**Most static and total pressures were measured using the Scanivalve Digital Signal Arrays (DSA) with 16 temperature-compensated piezoresistive sensors (accuracy ±0.05%). Absolute pressures were measured using a Mensor CPT 6100 transducer (Mensor LP, San Marcos, TX, USA) (accuracy ± 0.01% FS). Differential and absolute pressures across an ASME-standard venturi were captured with Rosemount 3051C transducers (accuracy 0.14% FS) to calculate mass flow. Stall onset was monitored with an Omega PX319 sensor (Omega Engineering, Inc., Norwalk, CT, USA) (response < 1 ms, accuracy ± 0.25%).

T-type thermocouples were read with an Agilent 34890A integrating voltmeter (Agilent Technologies, Inc., Santa Clara, CA, USA) (accuracy ± 0.9 °F). Cold junctions were measured with MAXIM DS1631U IC thermometers (Maxim Integrated, San Jose, CA, USA) (±0.9 °F) and refined with Omega RTD probes (±0.2 °F). For data acquisition National Instruments LabVIEW 2013 was used.

Total pressure rakes at 30° and 210° and total temperature rakes at 150° and 330° characterized impeller inlet flow. Downstream, static pressure taps verified flow uniformity, with 10 along the shroud and additional circumferential ports at 99% passage. Bleedlines at 60° and 240° allowed controlled flow extraction from the impeller backface.

Six CapaciSense capacitance probes were flush-mounted at two meridional positions (knee and exducer) and three circumferential locations (30°, 150°, 270°). The system measured real-time blade clearances through frequency modulation, with a maximum uncertainty of 4 × 10^−4^ in. Clearance measurements were synchronized with once-per-revolution signals for blade-specific monitoring.

**Figure 18:**
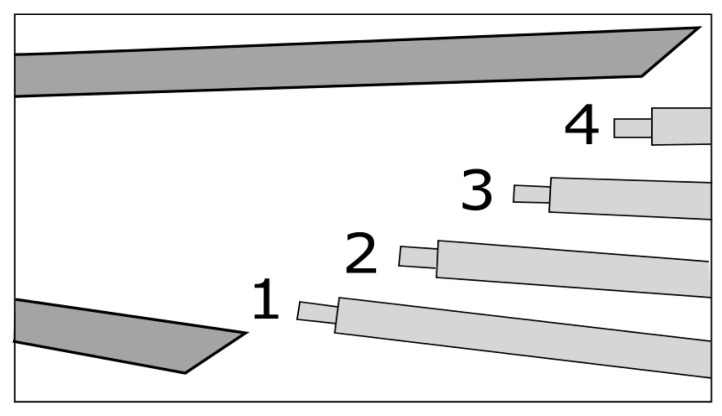
Diffuser exit total pressure rake nomenclature: wedge diffuser: 1: 11.9 pitch location (% vane passage); 2: 35.7; 3: 59.5; 4: 83.4; airfoil diffuser: 1: 3.39 pitch location (% vane passage); 2: 26.87; 3: 57.13; 4: 80.25 [78].

A front view of the compressor impeller and diffuser is depicted in Figure 17a, with the main instrumentation layout shown in Figure 17b. For this study, different pitch locations were analyzed, with their corresponding numbers relative to the diffuser passages presented in Figure 18.

**Influence on Results—**An airfoil diffuser was designed using CFD to improve stage efficiency and pressure recovery relative to a baseline wedge diffuser. Experimental tests at different impeller tip clearances showed the following:

- Tighter clearances reduced over-the-tip leakage, improving total pressure ratio, isentropic efficiency, and choking mass flow.
- Tip clearance had minimal impact on surge margin.

Compared to the wedge diffuser, the airfoil increased pressure recovery and diffuser effectiveness, though overall stage isentropic efficiency was slightly lower due to high losses in the deswirl region caused by manufacturing deviations.

The study highlights the critical role of advanced instrumentation for capturing detailed flow and structural phenomena and demonstrates that even localized improvements in diffuser design may be constrained by physical assembly and manufacturing tolerances. Precise component matching remains essential for achieving predicted performance gains.

#### 5.2.2. Optical/Velocity-Based Instrumentation

**A.**  **Purdue University High-Speed Centrifugal Compressor Facility**

The Purdue University High-Speed Centrifugal Compressor Facility is a state-of-the-art experimental platform designed to study detailed flow behavior within centrifugal compressor diffusers under high-speed, realistic operating conditions. The facility is powered by an Allison 250-C30G turboshaft engine (Allison Engine Company, Indianapolis, IN, USA), which drives a research compressor through a gear reduction system. The test compressor incorporates an advanced 50° backsweep impeller with 15 full and splitter blades, followed by 22 wedge-type diffuser vanes. The nominal operating speed of the compressor is 48,450 rpm, with a diffuser inlet-to-impeller exit radius ratio of 1.094.

**Instrumentation Setup—**The facility is equipped with a combination of steady-state and optical diagnostic systems. Conventional instrumentation includes steady temperature and pressure probes for determining rotational speed, mass flow rate, pressure ratio, and overall efficiency. The mass flow rate is computed using total and static pressure measurements from dual rakes located upstream of the test section, while the pressure ratio is determined using four three-headed total-pressure rakes positioned across four diffuser passages. Exit temperature measurements in the plenum complete the steady-state dataset. The compressor is throttled via a butterfly valve at the exit duct, and its rotational speed is adjusted by varying the turboshaft engine output.

In addition to conventional probes, the facility employs Particle Image Velocimetry (PIV) to capture planar velocity fields within the diffuser. The PIV system consists of a Solo PIV Nd:YAG laser (New Wave Research, Fremont, CA, USA) (532 nm wavelength), a Hi-Sense MKII CCD camera (Dantec Dynamics, Skovlunde, Denmark) with a Nikon Nikkor 35 mm lens, and Dantec Dynamics Flow Manager (v4.71) for synchronization and data processing. A Berkeley Nucleonics pulse delay generator and a laser tachometer trigger enable phase-locked data acquisition at various impeller–diffuser positions. Flow seeding is provided by a Topas ATM 210/H aerosol generator (Topas GmbH, Dresden, Germany) using diethylhexyl sebacate particles with a mean diameter of 0.25 μm, ensuring accurate tracking of flow structures up to 1% velocity error within the subsonic and transonic regions.

**Figure 19:**
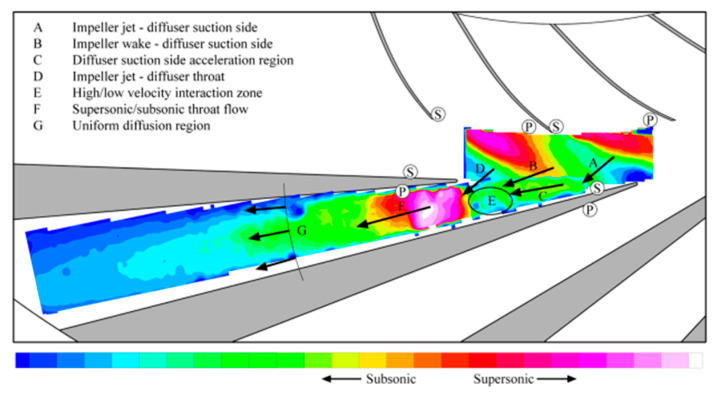
Impeller flow interaction with the diffuser [79].

**Influence on Results—**PIV measurements revealed that the diffuser flow field exhibits complex, three-dimensional structures, deviating substantially from idealized steady diffusion (Figure 19). The semi-vaneless-space acceleration region was found to be only weakly dependent on compressor loading, whereas the diffuser throat flow patterns were strongly influenced by load conditions. The PIV data enabled visualization of local Mach number distributions and flow recirculation zones, confirming strong circumferential non-uniformities and persistent momentum gradients downstream of the impeller.

**B.**  **NASA Glenn Research Center—Instrumentation for Active and Passive Flow Control in a Centrifugal Compressor Diffuser**

A series of controlled experiments were conducted at the NASA Glenn Research Center to investigate methods of extending the stable operating range of a high-speed centrifugal compressor. The test compressor, a scaled Rolls-Royce Allison design, was configured for a design pressure ratio of 4:1 at a corrected design speed of 21,789 rpm and a mass flow of 4.54 kg/s. The impeller contained 15 main blades and 15 splitter blades with 50° backsweep, discharging into a vane-island diffuser with 22 vanes.

**Instrumentation Setup—**The facility was comprehensively instrumented to capture both steady-state and transient performance characteristics during active flow control tests. The compressor was equipped with high-frequency response pressure transducers installed along the diffuser passage and vaneless space, enabling time-resolved monitoring of local pressure variations during surge and recovery cycles. These sensors were flush-mounted with the diffuser shroud surface and arranged circumferentially to construct spatio-temporal (X-T) pressure diagrams during instability events.

For the steady-state characterization, total and static pressure measurements were obtained at the compressor inlet and diffuser exit to determine the overall pressure ratio and efficiency. The compressor rig also featured injection and obstruction systems integrated directly into the diffuser shroud to assess both active air injection and passive blockage effects on flow stability.

**Figure 20:**
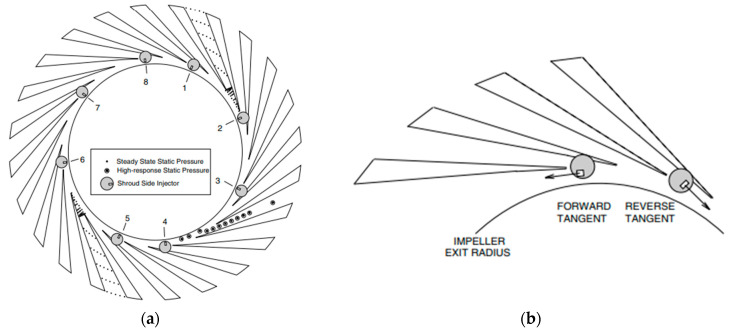
Centrifugal compressor test-rig instrumentation: (**a**) diffuser shroud instrumentation and injector locations; (**b**) shroud side injector orientation [80].

Eight injection nozzles were installed circumferentially on the shroud side of the diffuser, designed to deliver steady air jets into the vaneless region (Figure 20). The air supply was controlled via an external manifold that could either draw air from an external source or from recirculated discharge flow collected through Pitot pickups at the diffuser exit.

For the passive control configuration, steel tubes (10 mm diameter) were inserted through the same shroud openings, aligned with the local absolute velocity vector. Immersion depth was varied using shims, and the tube ends were capped to prevent mass flux, thereby acting as localized flow obstructions.

**Figure 21:**
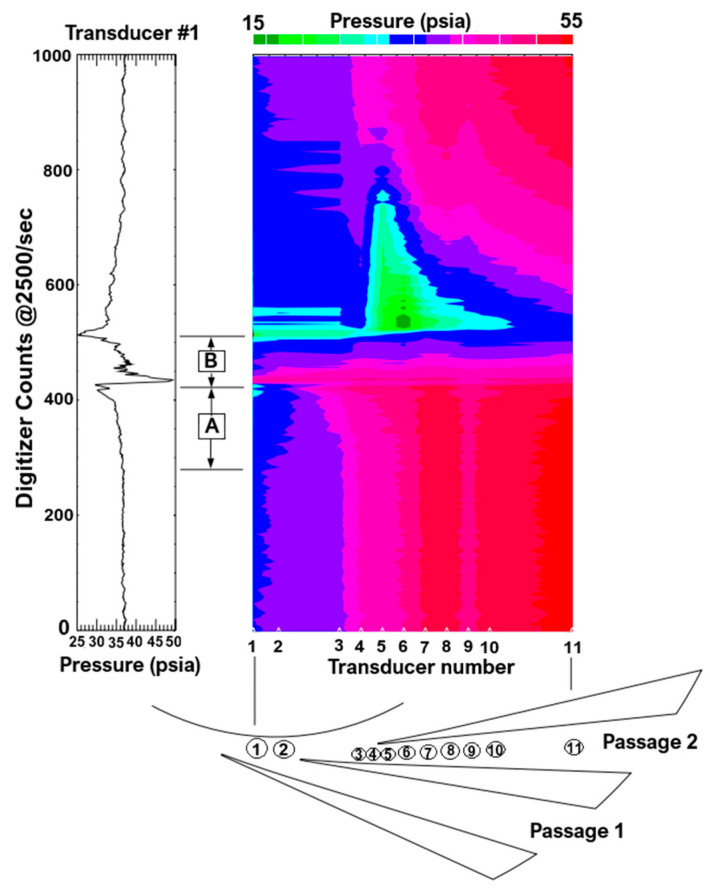
X-T Diagram of diffuser passage pressure through one surge cycle [80].

**Influence on Results—**Some results showing the X-T diagram of high-response pressure measurements collected in the diffuser during one surge cycle are presented in Figure 21. The figure also includes a pressure trace from the first vaneless space transducer.

The high-response pressure transducers provided detailed insight into the pressure-wave propagation during surge cycles and allowed construction of X-T diagrams that revealed the spatial evolution of unsteady pressure fronts through the diffuser. This instrumentation enabled researchers to correlate local unsteady pressure signatures with surge inception and recovery, a capability not achievable with steady probes alone.

The results showed that steady air injection through the diffuser shroud produced a 1.7-point increase in surge margin when supplied from within the compressor and enabled the compressor to recover from surge when air was supplied externally. In contrast, the insertion of capped obstruction tubes produced an even larger 6.5-point improvement in surge margin over the baseline configuration. These findings directly linked pressure-field measurements with the observed stability improvements, demonstrating how localized shroud instrumentation can be used to both monitor and actively influence diffuser flow behavior.

While the presented case studies illustrate the diversity of diffuser instrumentation strategies, several methodological limitations should be acknowledged. Many setups relied on sparse circumferential or radial distributions of probes, which limited the ability to resolve three-dimensional flow structures or identify localized separation zones; in several cases, probe placement decisions were driven by geometric accessibility rather than an optimized measurement strategy. High-frequency unsteady pressure sensors provided valuable insight into stall and surge dynamics, yet the accuracy of these measurements is constrained by thermal drift, mounting stiffness, and unknown local flow-angle effects—none of which were fully characterized in the original experiments. Optical methods such as PIV and LDV, although powerful, were restricted to isolated optical ports, and the results therefore represent only a subset of diffuser passages; seeding non-uniformity and laser reflections introduce additional bias that is difficult to quantify. In several industrial rigs, temperature measurements relied on slow-response thermocouples whose thermal inertia limits the reliability of transient surge detection. Furthermore, many of the reviewed studies do not explicitly justify why certain planes, frequencies, or sensor types were chosen or how their limitations influence the interpretation of flow phenomena. Collectively, these constraints highlight that the case-study results, while insightful, should be interpreted with caution, as the instrumentation used in each campaign captures only a partial representation of the highly unsteady flow field inside centrifugal compressor diffusers.

Looking ahead, several measurement trends appear particularly promising for advancing diffuser diagnostics beyond the limitations of current practice. In the authors view, the most significant step change will come from the maturation of miniaturized, high-bandwidth MEMS and conformal sensor arrays, which offer the potential for dense pressure-temperature mapping directly on diffuser walls with negligible blockage—something unattainable when using conventional probe-based systems. Parallel progress in fiber-optic pressure/temperature sensors, immune to electromagnetic interference, and capable of embedding inside rotating components, suggests new opportunities for rotor-stator interaction studies. On the optical side, high-speed PSP/TSP and volumetric PIV/BOS techniques are expected to become more accessible as illumination and camera technologies continue to shrink in size and cost, enabling more realistic in-rig applications rather than laboratory-only use. Another emerging direction is the integration of hybrid sensing architectures, combining unsteady pressure, thermal, vibration, and optical diagnostics into synchronized datasets that can feed digital twins and AI-assisted instability detection methods. Finally, the growing use of additive-manufactured instrumented components is anticipated, where embedded channels or sensor cavities are printed directly into diffusers or deswirl casings to achieve higher spatial resolution with minimal aerodynamic penalty. Taken together, these trends indicate a shift toward high-density, minimally intrusive, and data-fusion-ready sensing technologies that can support real-time health monitoring and substantially deepen our understanding of diffuser flow physics.

## 6. Analysis and Discussion

The experimental data collected from a range of centrifugal compressors, spanning laboratory research facilities and industrial applications, underscores the critical role of instrumentation type, placement, and measurement fidelity in understanding flow instabilities and surge phenomena.

A. Pressure-Based Monitoring

High-frequency pressure transducers consistently emerge as the primary diagnostic tool for detecting flow instabilities. Across all cases—from the DLR–Liebherr SSCC [68] to industrial high-speed turbochargers—dynamic pressure measurements with sampling rates of 20–200 kHz and resolutions down to 0.07 mbar allowed identification of stall precursors, surge onset, and rotating instabilities (RIs). For instance, the DLR–Liebherr SSCC facility demonstrated that distributed transducer arrays could detect mode-locked RI behavior near surge, while the sewage system compressor captured three-lobe rotating stall modes at low impeller speeds (~30.8% of design speed).

Placement is critical: circumferentially and radially distributed sensors provide detailed spatiotemporal mapping of pressure propagation and asymmetric instability modes. Industrial turbocharger tests revealed that pressure probes could detect small-scale flow perturbations, while integration with fast-response thermocouples enabled robust multi-parameter criteria for surge detection.

These results highlight that pressure-based instrumentation, when densely deployed and synchronized with data acquisition systems (e.g., DEWEsoft or NI DAQ), enables both detection of transient instabilities and validation of predictive computational models.

B. Temperature and Acoustic/Vibro-Acoustic Measurements

Fast-response thermocouples complement pressure measurements by providing sensitive indicators of surge onset. In high-speed turbochargers, temperature variance exhibited a clearer signature of surge than dynamic pressure alone, demonstrating the value of multi-parameter analysis.

Acoustic and vibration instrumentation, applied in two-stage electric fuel-cell compressors, extended detection capabilities to rotor-induced phenomena. Microphones and triaxial accelerometers, when sampled at 48 kHz, could predict rotating stall 1.5–3 s before onset. Spectral analyses (STFT/PSD) identified coupling between structural vibrations and flow instabilities, offering early-warning capabilities in industrial applications where optical access is limited.

C. Optical/PIV Techniques

Planar and digital particle image velocimetry (PIV/DPIV) provided spatially resolved insights into 3D flow structures, recirculation zones, and modal surges. Purdue University [73] and NASA Glenn [74] facilities demonstrated that phase-locked PIV, combined with high-speed CCD imaging, could visualize complex diffuser flows and transonic inlet effects, with measurement uncertainty below 1%.

The integration of optical measurements with high-frequency pressure transducers allowed direct correlation of local velocity fields with global pressure signatures. This is particularly valuable in research environments where validation of numerical models requires time-resolved 3D flow data.

Across both research and industrial compressors, several key trends emerge:

- Instrumentation fidelity and placement: dense, high-frequency transducer networks, particularly with circumferential and radial coverage, provide superior resolution of propagating instabilities. Strategic placement in diffuser passages and impeller vaneless spaces captures critical precursor events.
- Multi-parameter integration: combining pressure, temperature, PIV, and acoustic measurements significantly enhances detection accuracy. Synchronized systems (IRIG-B or phase-locked) allow precise correlation between flow field dynamics, pressure fluctuations, and structural response, enabling robust predictive model validation.
- Flow control evaluation: In NASA Glenn experiments, distributed pressure sensors directly quantified the impact of active (air injection) and passive (capped tubes) flow control on surge margin, demonstrating the potential for real-time monitoring and optimization of compressor stability.
- Challenges: optical methods, while highly informative, are constrained by seeding uniformity, optical access, and large data volumes. Acoustic measurements can be influenced by environmental noise and require careful calibration to interpret instability signals accurately.

The comparative analysis provided in Table 3 emphasizes that a multi-modal instrumentation approach is essential for fully characterizing centrifugal compressor dynamics:

- High-speed pressure transducers provide primary detection of stall and surge, enabling frequency-domain analyses for model validation.
- Thermocouples, microphones, and accelerometers enhance early warning capabilities for rotating instabilities.
- Optical methods provide spatial validation of simulations and reveal three-dimensional flow features inaccessible via point measurements.
- Integration and synchronization are fundamental, as isolated measurements they cannot fully capture coupled flow–structure interactions.

Overall, the findings highlight that experimental insights are highly dependent on instrumentation quality, placement, and integration. Research facilities with combined PIV, pressure, and acoustic measurements achieve superior fidelity in mapping flow instability evolution, whereas industrial installations benefit from practical, high-speed point measurements that can inform predictive maintenance and real-time monitoring.

Table 4 summarizes and compares various diagnostic and analytical approaches used to study diffuser flow phenomena in compressors, highlighting their configurations, capabilities, and practical considerations.

Key Comparative Insights

A. Sensor Types and Roles

- Pressure transducers: Essential for mapping transient instabilities and detecting stall/surge precursors (Cases 1, 2, 4).
- Thermocouples: Fast-response thermocouples were especially useful for surge detection (Case 4).
- Acoustic and vibration sensors: Enabled indirect detection of instabilities and provided predictive capabilities (Case 3).
- Optical methods (PIV): Allowed direct visualization of complex flow fields and shock structures (Case 1, 2).

B. Data Acquisition Rates

- High-frequency sampling (≥20 kHz) is critical to capture blade-passing frequencies and transient flow phenomena.
- Optical PIV requires phase-resolved synchronization to reconstruct instantaneous flow fields.

C. Placement and Coverage

- Circumferential arrays in impeller and diffuser regions enhance the ability to track rotating instabilities (Cases 2, 4).
- Strategic microphone and accelerometer placement captures acoustic precursors of surge in remote locations (Case 3).
- PIV measurements at inlet and diffuser exit capture spatio-temporal evolution of velocity fields (Cases 1, 2).

D. Insights Enabled

- Pressure transducers + thermocouples allow quantitative surge/stall detection via SD, variance, and PSD analyses.
- Vibro-acoustic measurements enable early stall detection and validation of predictive models.
- PIV captures complex flow structures like shock formation, diffuser flow non-uniformities, and circumferential momentum variations.

E. Limitations

- Microphones are sensitive to mechanical vibrations and require filtering.
- PIV is limited in shock resolution due to particle “smearing” and optical access constraints.
- High-density transducer arrays require careful calibration to ensure phase synchronization.

Table 5 presents various instrumentation methods used to study diffuser flow and performance, along with their configurations, key findings, advantages, and limitations.

## 7. Conclusions

This review demonstrates that accurate characterization of diffuser flow behavior relies heavily on the appropriate selection, placement, and synchronization of instrumentation. Across all surveyed facilities, high-frequency pressure transducers consistently proved essential for resolving stall precursors, rotating instabilities, and surge cycles, providing the most direct insight into unsteady flow mechanisms. Complementary temperature measurements—particularly fast-response thermocouples—were shown to be equally valuable for tracking thermal fluctuations associated with instability and, in several studies, served as the most reliable surge indicators. Optical diagnostics, including PIV, LDV, and PSP/TSP, offer unmatched spatial details and continue to bridge critical gaps in understanding complex diffuser aerodynamics. Although their applicability in industrial environments remains limited, these methods provide indispensable validation data for CFD, digital twins, and reduced-order models. Acoustic and vibration measurements further enrich diffuser monitoring by revealing dynamic coupling between aerodynamic disturbances and structural responses, enabling early detection of abnormal operating conditions.

Based on the surveyed techniques, several instrument classes emerge as the most effective for diagnosing key diffuser phenomena. For surge detection, the most reliable indicators remain high-bandwidth wall-pressure transducers and fast-response thermocouples, which consistently capture the rapid rise in low-frequency pressure and temperature fluctuations preceding full surge. Among these, miniature piezoresistive sensors and microsecond-response thermocouples demonstrate superior sensitivity to deep-surge onset compared with microphones or slower thermal probes. For rotating stall, circumferential arrays of unsteady pressure sensors positioned in the vaneless space or at the diffuser inlet are recommended, as they resolve modal structures and propagation speeds that cannot be detected by global vibration or acoustic signals alone. These arrays should be sampled at ≥20–50 kHz to capture the sub-harmonic content associated with stall precursors. Finally, flow separation and diffuser loss mechanisms are best resolved using a combination of dense static-pressure mapping and angle-measuring multi-hole probes at the throat and mid-passage, complemented—where optical access permits—by PIV or time-resolved PSP/TSP to reveal boundary-layer development and recirculation pockets. In practice, the most robust approach is hybrid: unsteady wall-pressure and temperature sensors for stability monitoring, probe-based measurements for passage-level flow reconstruction, and optical methods for high-resolution characterization.

Recent advances in data-driven modeling suggest that neural networks can substantially enhance the quality and interpretability of diffuser measurements. Neural networks trained on synchronized pressure, temperature, vibration, and optical datasets can reconstruct high-resolution flow fields from sparse inputs, effectively acting as virtual sensors capable of inferring local incidence, swirl, and separation. For surge and rotating-stall monitoring, recurrent architectures such as LSTM networks have demonstrated the ability to identify instability precursors several cycles earlier than traditional threshold-based approaches by learning characteristic temporal patterns in unsteady pressure signals. Convolutional neural networks (CNNs) can likewise denoise PSP/TSP images, compensate for illumination issues, and enhance spatial resolution. When integrated within hybrid sensing architectures, neural networks enable real-time anomaly detection, adaptive data fusion, and improved uncertainty estimation under rapidly changing operating conditions. These developments complement—not replace—conventional instrumentation, offering a pathway toward predictive, high-fidelity diffuser monitoring.

Across industrial, laboratory, and high-speed research compressors, the case studies confirm that no single measurement technique is sufficient. Instead, hybrid instrumentation architectures combining steady probes, high-bandwidth sensors, and optical diagnostics provide the most robust and interpretable datasets. The review also underscores the continuing need for advances in sensor miniaturization, higher bandwidth, improved calibration methodologies, and tighter integration with data-driven analysis. Collectively, these improvements are essential for enabling real-time diffuser monitoring, enhancing compressor reliability, and strengthening surge resistance in next-generation centrifugal compressor systems.

## Figures and Tables

**Table 1:** Indicative uncertainty budget for inlet/duct instrumentation AIP.

Quantity	Main Contributions	Typically Expanded Uncertainty
Total pressure ($P_{0}$)	Transducer calibration, resolution, temperature drift, rake blockage, port purging	±0.2–0.5% of reading
Static pressure (*p*)	Tap location error, line losses, transducer cal., temp. effects	±0.2–0.5% of reading
Total temperature ($T_{0}$)	Probe recovery factor *r*, radiation/conduction, time lag, calibration	±0.5–1.0 K
Axial velocity ($W_{x}$)	From $P_{0}-p$ relation, EOS ρ and angle misalignment	±1–3%
Tangential velocity ($W_{\theta }$)	Multi-hole calibration map, alignment, port bias	±2–4%
Radial velocity $\left(W_{r}\right)$	Multi-hole calibration, probe stem interference	±3–5%
Yaw (swirl) angle (α)	Multi-hole map fit, zeroing, alignment	±0.5–1.5°
Radial angle (β)	As above, low signal levels	±0.7–2.0°
Circumferential distortion (${DC}_{\theta }$)	Sector averaging, weighting (mass-flux), rake blockage, sparse sampling	±0.01–0.03 (abs.)
Radial distortion (${DR}_{r}$)	Ring averaging, near-wall bias, sparse sampling	±0.01–0.03 (abs.)
Turbulence intensity (*Tu*)	Sensor bandwidth, noise floor, sampling duration, seeding (PIV/LDV)	±0.2–0.5% (abs.)
Boundary layer thickness (δ)	Probe positioning, near-wall resolution, interpolation	±0.5–1.0 mm
Shape factor (*H)*	Profile integration, near-wall accuracy	±0.10–0.20
Mass-flux weighting ($w_{i}$)	Area partitioning	±0.5–1.5%
Probe positioning (x, r, θ)	Traverse repeatability, backlash, fixturing	±0.2–0.5 mm (radial), ±0.5–1.0° (θ)
Acquisition bandwidth	Anti-aliasing, sampling rate, filter phase lag	Specified, not a%

**Table 2:** Summary of sensor types and their positioning on a centrifugal compressor diffuser.

Sensor Type	Positioning	Purpose
Piezoelectric/MEMS accelerometers	Diffuser casing, near vanes, hub, or support structures	Mechanical vibration monitoring
Laser Doppler Vibrometer	Non-contact on blades or casing	Lab vibration analysis
Microphones	External casing, anechoic chambers	Aerodynamic noise detection
Pressure transducers	Flush with walls near vanes, hub, and shroud	Pressure pulsations and flow instabilities
Acoustic beamforming arrays	Surrounding diffuser	Noise source localization

**Table 3:** Summary of diffuser measurement techniques and their main shortcomings.

Method	Key Advantages	Main Shortcomings/Outdated Aspects
Pitot/Kiel probes	Simple, robust, standardized; good for mean pressure fields	Intrusive; limited frequency response; blockage effects; yaw sensitivity; unsuitable for fast unsteady phenomena
Static taps and tubing systems	Accurate mean pressures; easy integration	Dynamic distortion, resonance, purge issues; limited to steady or low-frequency measurements
Multi-hole probes	Measures pressure + flow angles; essential for swirl mapping	Calibration-intensive; alignment-sensitive; fragile at high T; limited bandwidth
Hot-wire/hot-film anemometry	High temporal resolution for turbulence	Fragile; strong temperature constraints; intrusive; outdated for industrial compressors
Thermocouples/RTDs	Widely available; reliable for steady-state conditions	Slow response (most types); conduction/radiation bias; inadequate for surge dynamics
MEMS pressure/temperature sensors	High bandwidth; miniaturized; promising for rotors	Not yet robust for long-term high T operation; drift; packaging complexity
PSP/TSP	Global high-density fields; excellent for research	Requires optical access; temperature/illumination corrections; limited industrial applicability
LDV/PIV	High-resolution, non-intrusive velocity fields	Needs optical windows; seeding issues; costly; not feasible for in situ machinery
High-speed unsteady pressure arrays	Essential for stall/surge detection; kHz–MHz bandwidth	Local-only; cannot measure vector fields; sensitive to mounting and thermal gradients
Acoustic and vibration sensors	Good for instability detection; easy to integrate	Indirect measurements; require filtering; cannot resolve local flow structures

**Table 4:** Comparative analysis of centrifugal compressor instrumentation and experimental insights.

Case/Facility	Compressor Type and Specs	Instrumentation	Placement/Configuration	Sampling and Measurement Specs	Key Results/Observations
DLR–Liebherr SSCC [70]	Single-stage, 15 unshrouded backswept blades, vaneless diffuser, asymmetric volute; aircraft AC system	19 Kulite XCE-062 pressure transducers; Dewetron 808 DAQ; IRIG-B timing; TR-PIV	Flush-mounted in impeller casing, 3 meridional positions; PIV upstream (3.0r1) and downstream (1.1r2)	Pressure: 0.07–0.12 mbar resolution, 200 kHz, 150 kHz BW; PIV: 54 kHz frame rate, 16–64 px interrogation windows	Detection of rotating instabilities (RI); mode-locked behavior near surge; low-frequency coherence between pressure and velocity
Industrial Centrifugal Compressor (Sewage System) [71]	Single-stage, vaneless diffuser, 180 kW, 18,300 rpm, PR max 1.88	Kulite XCE-093 dynamic pressure transducers, thermocouples; NI 9215 + DAQ-9178	7 radial locations, circumferentially distributed across impeller + diffuser	Pressure: 0–300 kPa, 0.05% accuracy, 20 kHz sampling	Three-lobe rotating stall mode at ~30.8% impeller speed; tip leakage amplified instability
High-Speed Turbocharger Centrifugal Compressor [72]	Single-stage, vaneless diffuser, high-speed (185,000 rpm)	Kulite pressure probes, fast-response thermocouples, ICP microphones; DEWEsoft DAQ	P1/T1 at impeller inlet (280°/200°), P2/T2 mid-diffuser (50°/110°); microphones near shroud (S1) and inlet duct (S2)	Pressure and temperature: 50 kHz; microphones: 50 kHz, low-pass 15% shaft freq	SD slope of dynamic pressure rises near surge; temperature variance most effective for surge detection; microphones less reliable unfiltered
CSTAR—Purdue University [73]	Low-specific-speed axi-centrifugal compressor; design corrected speed 22,500 RPM; PR ≈ 3; aeroengine research stage	T-type thermocouples; total temperature rakes; Scanivalve DSAs; LDV system for impeller-exit flow	Temperature rakes at impeller inlet, diffuser inlet, and turn-to-axial exit; pressure taps along impeller, diffuser, and turn-to-axial (spanwise + circumferential); LDV optical access at impeller exit	Steady + transient temperature/pressure sampling via Scanivalve/DAQ; LDV for velocity field; spatially distributed static/total pressure measurements	Incidence increases as flow reduces; positive incidence improves diffuser performance; leading-edge vanes highly loaded near surge; spike-type stall originates in vaneless space; unsteady loading governs vane failure
Two-Stage Electric Compressor for Fuel Cells [74]	Two-stage, high-speed, >100 kW	G.R.A.S. 40PH microphones, KISTLER triaxial accelerometers; HEAD ArtemiS SUITE	Microphones: first/second stage inlets/outlets, 1 m distance, 45° offset; accelerometers on motor, ducts, compressor surfaces	Microphones: 20–20,000 Hz, 48 kHz sampling; accelerometers: 20–10,000 Hz, 48 kHz	SPL growth (20–40% RSF) predicted rotating stall 1.5–3 s in advance; surge model validated (<1.95% error)
Purdue SSCC Facility [76]	Honeywell single-stage centrifugal compressor for diffuser/stage performance studies	Total pressure and temperature probes; static taps; 2 inlet thermocouples; fast-response pressure transducers	Probes at stations 0 (inlet), 5 (diffuser exit), 6 (deswirl exit); static taps along impeller–diffuser–deswirl; thermocouples 180° apart upstream of impeller; unsteady sensors distributed through flow path	Steady-state pressure/temperature measurements; high-frequency unsteady pressure acquisition for stall/surge detection	Fast-response sensors distinguished spike-type (deep) surge in subsonic and supersonic regimes and modal-type (mild) surge near transonic inlet Mach; correlation of static pressure rise with instability onset;
NASA Glenn—Rolls-Royce Allison Centrifugal Compressor [77]	Single-stage, impeller + vaned diffuser; 21,789 rpm, PR 4:1, 4.54 kg/s	High-frequency dynamic pressure transducers; DPIV with particle seeding	Pressure: vaneless space + diffuser, circumferential; DPIV: optical access via periscope, four diffuser passages	Pressure: real-time monitoring of BPF; DPIV: instantaneous velocity fields	Stall inception location speed-dependent (impeller ≤ 70% speed, diffuser at design); visualized flow reversal and surge cycles
CSTAR—Purdue University (Airfoil vs. Wedge Diffuser Study) [78]	Centrifugal stage, design corrected speed 22,500 rpm; engine-representative Mach numbers; ABB AC motor driveline	Scanivalve DSA pressure arrays; Mensor CPT 6100 absolute transducer; Rosemount 3051C DP/absolute sensors; Omega PX319 stall sensor; T-type thermocouples; Agilent 34890A voltmeter; MAXIM DS1631U and Omega RTD cold junction sensors; CapaciSense blade-clearance system	Total pressure rakes at 30°/210° and temperature rakes at 150°/330°; static taps along shroud + circumferential taps at 99% span; bleedlines at 60°/240°; 6 capacitance probes at knee and exducer (30°, 150°, 270°)	Pressure accuracy: ± 0.05% (DSA), ±0.01% FS (Mensor), 0.14% FS (Rosemount); stall sensor < 1 ms response; thermocouples ± 0.9 °F; clearance uncertainty 4 × 10^−4^ in.; LabVIEW DAQ	Airfoil diffuser increased pressure recovery; tighter tip clearance improved PR/efficiency with little surge-margin effect; deswirl losses limited overall efficiency; diffuser benefits constrained by manufacturing/assembly tolerances
Purdue University High-Speed Centrifugal Compressor Facility [79]	Single-stage Honeywell compressor; 48,450 rpm, 50° backsweep impeller, 22 diffuser vanes	Steady and fast-response pressure transducers; total/station temperature probes; PIV	Pressure/temperature at key stations (inlet, diffuser exit, deswirl exit); PIV in diffuser	PIV: 2D planar velocity; high-speed CCD; laser-based seeding; measurement uncertainty < 1%	Spike-type deep surge at subsonic/supersonic inlet, modal mild surge at transonic inlet; 3D flow recirculation captured
NASA Glenn—Active and Passive Flow Control [80]	Rolls-Royce Allison design; impeller + 22-vane diffuser; 21,789 rpm, PR 4:1	High-response pressure transducers; injection and obstruction system for flow control	Transducers along diffuser + vaneless space, circumferentially; injection nozzles and steel tubes in shroud	Real-time X-T pressure diagrams	Air injection improved surge margin 1.7 points; capped tubes increased margin 6.5 points; pressure-wave evolution tracked

**Table 5:** Diagnostic techniques for characterizing diffuser flow behavior.

Instrumentation Method	Test Configuration	Key Findings	Advantages	Limitations
Dynamic Pressure Sensors (e.g., Kulite XCE-093)	Embedded in casing surface near tip clearance; synchronized data acquisition	Captures real-time dynamic pressure fluctuations, aiding in the analysis of unstable flow structures like stall and surge	High-frequency response, precise spatial resolution	Requires advanced data acquisition systems; sensitive to installation alignment
Hot-Wire Anemometry	Positioned at various diffuser locations to measure velocity profiles	Provides detailed velocity measurements, useful for identifying flow separation and vortex formation	High spatial and temporal resolution	Sensitive to temperature and contamination; requires calibration
Laser Doppler Velocimetry (LDV)	Non-intrusive measurement across diffuser cross-sections	Offers accurate velocity vector fields, beneficial for detailed flow analysis	Non-intrusive, high spatial resolution	Expensive equipment; complex data interpretation
Computational Fluid Dynamics (CFD) Simulations	Numerical modeling of diffuser geometry and flow conditions	Predicts flow behavior, pressure recovery, and identifies potential stall zones	Cost-effective for design iterations; detailed flow insights	Requires validation with experimental data; computationally intensive
Performance Test Rigs (e.g., Venturi flowmeter, Pitot tubes, thermocouples)	Full-scale compressor testing with controlled inlet and outlet conditions	Measures pressure ratios, flow rates, and temperatures to evaluate diffuser efficiency	Provides real-world performance data; comprehensive analysis	High setup costs; potential for measurement errors due to environmental factors

## Data Availability

The original contributions presented in this study are included in the article material. Further inquiries can be directed to the corresponding author.

## Abbreviations

The following abbreviations are used in this manuscript:

API	American Petroleum Institute
ASME	American Society for Mechanical Engineers
BPF	blade passing frequencies
CFVN	critical-flow Venturi nozzles
CFD	Computational Fluid Dynamics
CSTAR	Centrifugal Stage for Aerodynamic Research
DSA	Digital Sensor Arrays
DPIV	Digital Particle Image Velocimetry
FFT	Fast Fourier Transform
ISO	International Stadardisation Organisation
LDV	Laser Doppler Vibrometer
ODS	operational deflection shape
PIV	Particle Image Velocimetry
SSCC	single-stage centrifugal compressor
STFT	Short-Time Fourier Transform
TSP	temperature sensitive paints
